# Comparison of the National Early Warning Score (NEWS) and the Modified Early Warning Score (MEWS) for predicting admission and in-hospital mortality in elderly patients in the pre-hospital setting and in the emergency department

**DOI:** 10.7717/peerj.6947

**Published:** 2019-05-16

**Authors:** Toshiya Mitsunaga, Izumu Hasegawa, Masahiko Uzura, Kenji Okuno, Kei Otani, Yuhei Ohtaki, Akihiro Sekine, Satoshi Takeda

**Affiliations:** 1 Department of Emergency Medicine, Jikei University School of Medicine, Tokyo, Japan; 2 Centre for Preventive Medical Sciences, Chiba University, Chiba, Japan

**Keywords:** National early warning score, Modified early warning score, Elderly, Pre-hospital, Admission, Mortality, Risk score, ICU, EMS, Japan

## Abstract

The aim of this study is to evaluate the usefulness of the pre-hospital National Early Warning Score (pNEWS) and the pre-hospital Modified Early Warning Score (pMEWS) for predicting admission and in-hospital mortality in elderly patients presenting to the emergency department (ED). We also compare the value of the pNEWS with that of the ED NEWS (eNEWS) and ED MEWS (eMEWS) for predicting admission and in-hospital mortality. This retrospective, single-centre observational study was carried out in the ED of Jikei University Kashiwa Hospital, in Chiba, Japan, from 1st April 2017 to 31st March 2018. All patients aged 65 years or older were included in this study. The pNEWS/eNEWS were derived from seven common physiological vital signs: respiratory rate, peripheral oxygen saturation, the presence of inhaled oxygen parameters, body temperature, systolic blood pressure, pulse rate and Alert, responds to Voice, responds to Pain, Unresponsive (AVPU) score, whereas the pMEWS/eMEWS were derived from six common physiological vital signs: respiratory rate, peripheral oxygen saturation, body temperature, systolic blood pressure, pulse rate and AVPU score. Discrimination was assessed by plotting the receiver operating characteristic (ROC) curve and calculating the area under the ROC curve (AUC). The median pNEWS, pMEWS, eNEWS and eMEWS were significantly higher at admission than at discharge (*p* < 0.001). The median pNEWS, pMEWS, eNEWS and eMEWS of non-survivors were significantly higher than those of the survivors (*p* < 0.001). The AUC for predicting admission was 0.559 for the pNEWS and 0.547 for the pMEWS. There was no significant difference between the AUCs of the pNEWS and the pMEWS for predicting admission (*p* = 0.102). The AUCs for predicting in-hospital mortality were 0.678 for the pNEWS and 0.652 for the pMEWS. There was no significant difference between the AUCs of the pNEWS and the pMEWS for predicting in-hospital mortality (*p* = 0.081). The AUC for predicting admission was 0.628 for the eNEWS and 0.591 for the eMEWS. The AUC of the eNEWS was significantly greater than that of the eMEWS for predicting admission (*p* < 0.001). The AUC for predicting in-hospital mortality was 0.789 for the eNEWS and 0.720 for the eMEWS. The AUC of the eNEWS was significantly greater than that of the eMEWS for predicting in-hospital mortality (*p* < 0.001). For admission and in-hospital mortality, the AUC of the eNEWS was significantly greater than that of the pNEWS (*p* < 0.001, *p* < 0.001), and the AUC of the eMEWS was significantly greater than that of the pMEWS (*p* < 0.01, *p* < 0.05). Our single-centre study has demonstrated the low utility of the pNEWS and the pMEWS as predictors of admission and in-hospital mortality in elderly patients, whereas the eNEWS and the eMEWS predicted admission and in-hospital mortality more accurately. Evidence from multicentre studies is needed before introducing pre-hospital versions of risk-scoring systems.

## Introduction

The life expectancy in Japan is 80.98 years for men and 87.14 years for women; the life expectancy is the second highest in the world for men and the highest in the world for women (World Health Statistics, 2017). The proportion of people older than 65 years was 23.0% in 2010—the highest in the world—and is expected to reach 29.1% by 2020 (Lee et al., 2015). The number of patients older than 65 years presenting to the emergency department (ED) is also increasing in parallel with the increase in the elderly population. A study from the United States reported that elderly patients comprised 40–50% of all people presenting to the ED (Lee et al., 2018).

Several risk-scoring systems have been established to identify the risk of catastrophic deterioration and death in-hospital inpatients. The National Early Warning Score (NEWS) was developed in 2012 in the United Kingdom by the NEWS Development and Implementation Group on behalf of the Royal College of Physicians (Royal College of Physicians London, 2012). The Modified Early Warning Score (MEWS) was validated in 2001 in the United Kingdom as a bedside tool to identify patients at risk for catastrophic events, including death (Subbe et al., 2001).

Several studies have explored the association between these risk scores and hospital admission. The findings suggest that these risk scores could also be used as triage tools to identify patients requiring admission to hospital (Subbe et al., 2001; Burch, Tarr & Morroni, 2008; Cei, Bartolomei & Mumoli, 2009).

Most studies focused on scores that were measured in the hospital or the ED, and only a few studies evaluated the strong relation between pre-hospital NEWS (pNEWS) and in-hospital mortality or admission to the critical care unit (Abbott et al., 2018; Hoikka, Silfvast & Ala-Kokko, 2018). Our previous study showed that the abbreviated NEWS, which excludes respiratory rate, had moderate value for predicting admission and in-hospital mortality in elderly patients. However, this study had many limitations, such as selection bias and seasonal bias, and further studies in the pre-hospital setting are needed (Mitusnaga et al., 2018). There was no study comparing several risk-scoring systems in the pre-hospital setting, and it is still unclear which risk-scoring system is superior as a triage tool for admission and to predict in-hospital mortality of elderly patients who present to the ED by ambulance.

Only one study showed that the NEWS at admission was more strongly associated with escalation to death or critical care treatment than the pNEWS (Abbott et al., 2018). However, this study had several limitations, such as small sample size and short duration, and it is unclear whether pre-hospital early warning scores are superior to those from the ED.

The aim of the present study is to evaluate the value of pre-hospital early warning scores for predicting admission and in-hospital mortality in patients older than 65 years who present to the ED, by comparing the ED NEWS (eNEWS) and the ED MEWS (eMEWS).

## Materials and Methods

### Study design

This retrospective, single-centre observational study was carried out during 1 year in the ED of a university hospital in Japan to evaluate the value of the pNEWS and the pre-hospital MEWS (pMEWS) for predicting admission and in-hospital mortality in patients older than 65 years who were presented to the ED by ambulance.

### Study setting and population

In Japan, EDs are grouped into three categories. Primary emergency medical institutions treat patients with mild conditions and walk-in patients. Secondary emergency medical institutions treat patients with mild or moderate conditions that might require hospitalisation. Tertiary emergency medical institutions treat or resuscitate seriously ill patients who have suffered from multiple trauma, shock or cardiopulmonary arrest (Tanigawa & Tanaka, 2006). The present study was carried out between 1st April 2017 and 31st March 2018 at Jikei University Kashiwa Hospital, a tertiary emergency medical institution. The hospital is located in Kashiwa City in Chiba Prefecture. It has 664 beds, and about 8,500 patients present to the ED annually. We accept about 5,000 patients coming to the ED by ambulance annually. The population of Kashiwa City is about 415,000, and about 105,000 (25.3%) of the population is older than 65 years. People with disease or trauma call the fire department command centre (119), and the centre informs them of the emergency medical services (EMS) that are nearest to them. When the EMS personnel reach the patient, they gather information about the patient, including vital signs, only once, and judge the triage level. The EMS then calls the proper emergency institution and provides all the patient information. In our ED, a chief nurse receives the call and asks the ED doctors to accept the patient. All patients older than 65 years who presented to the ED by ambulance during the study period were included in this study. Patients arrested before arrival at the hospital and patients transferred to other hospitals from the ED were excluded from the study.

### Data sources and measurements

When the EMS arrive at our ED, the chief nurse first evaluates the severity of the case. Then, the patient is guided to the appropriate emergency room according to the severity. The junior and senior emergency medicine residents see all the patients who present to our ED, and the emergency physician takes over the patient’s treatment and follow-up. During this process, all of the patient’s data, including pre-hospital and ED vital signs, are recorded in electrical medical records by the nurses. We obtained the pre-hospital vital signs and the first vital signs just after arrival at the ED. The patients were followed up until discharge or death for a maximum of 28 days. Data on the patient’s discharge from the ED, admission to a ward, admission to the intensive care unit (ICU) and in-hospital mortality were recorded. Diagnostic categories were based on the International Classification of Diseases-10 and classified as (1) Trauma, (2) Neurology, (3) Pulmonology, (4) Cardiology, (5) Gastroenterology, (6) Endocrinology, (7) Nephrology/Urology, (8) Haematology, (9) Collagen disease, (10) Otolaryngology, (11) Gynaecology, (12) Dermatology, (13) Ophthalmology, (14) Psychology, (15) Toxicology and (16) Others.

We were able to obtain complete information on the patients’ age, gender, diagnostic category, length of stay in the ED, disposition and in-hospital mortality, but we could not obtain data on vital signs for all of the patients. The reasons for the missing data were that the EMS crew decided to give priority to transportation because the patient’s condition was extremely critical; the ED was overcrowded and the nurses were unable to input the patient’s information in the medical record, and some patients, such as those directed to otolaryngology, went directly to the treatment room. In the present study, missing values were not excluded, and substitution was made using multiple imputation analysis because transportation was prioritised in only a small number of cases, and most of the missing values occurred because the nurses were unable to input the data for vital signs due to overcrowding of the ED.

The pNEWS/eNEWS and pMEWS/eMEWS were calculated using the recorded physiological parameters of the patients. The pNEWS/eNEWS were derived from seven common physiological vital signs: respiratory rate, peripheral oxygen saturation, the presence of inhaled oxygen parameters, body temperature, systolic blood pressure, pulse rate and AVPU (Alert, responds to Voice, responds to Pain, Unresponsive) score. The scores vary between 0 and 3 for each parameter (Table 1). The pNEWS/eNEWS totals range from 0 to a maximum of 20. The pMEWS/eMEWS were derived from six common physiological vital signs: respiratory rate, peripheral oxygen saturation, body temperature, systolic blood pressure, pulse rate and AVPU score. The scores vary between 0 and 3 for each parameter (Table 2). The pMEWS/eMEWS totals range from 0 to a maximum of 14.

**Table 1:** National Early Warning Score (NEWS).

	3	2	1	0	1	2	3
Respiratory rate (bpm)	≤8		9–11	12–20		21–24	≥25
Oxygen saturation (%)	≤91	92–93	94–95	≥96			
Inhaled oxygen		Yes		No			
Temperature (°C)	≤35.0		35.1–36.0	36.1–38.0	38.1–39.0	≥39.1	
Systolic blood pressure (mmHg)	≤90	91–100	101–110	111–219			≥220
Pulse rate (bpm)	≤40		41–50	51–90	91–110	111–130	≥131
AVPU				A			V, P, or U

**Note:**

AVPU; A, alert; V, to voice; P, to pain; U, to unresponsive; bpm, beats or breaths per minute.

**Table 2:** Modified Early Warning Score (MEWS).

	3	2	1	0	1	2	3
Respiratory rate (bpm)		≤8	9	10–18	19–20	21–29	≥30
Oxygen saturation (%)	≤91	92–93	94–95	≥96			
Temperature (°C)		≤35.0		35.1–38.4		≥38.5	
Systolic blood pressure (mmHg)	≤70	71–80	81–100	101–199		≥200	
Pulse rate (bpm)		≤39	40–50	51–100	101–110	111–129	≥130
AVPU				A	V	P	U

**Note:**

AVPU; A, alert; V, to voice; P, to pain; U, to unresponsive; bpm, beats or breaths per minute.

The AVPU score was derived from the GCS as follows: *A* = 14–15, *V* = 9–13, *P* = 4–8, *U* = 3.

The patients were divided into three groups: those who were discharged from the ED, those who were admitted to a ward and those who were admitted to the ICU. The intergroup differences in all the parameters and the scores during the stay in the ED were also evaluated.

### Statistical analysis

Continuous variables were described as medians and interquartile ranges and were compared by Student’s *t*-test and Mann–Whitney *U*-test. Categorical variables were described as numbers and percentages and were compared by Pearson’s χ^2^ test. Analysis of variance was used to test differences among the three groups. Receiver operating characteristic analysis and the AUC were used to evaluate the predictive value of the pNEWS and pMEWS for admission and in-hospital mortality. Confidence intervals (CIs) around the AUC were calculated using bootstrap resampling methods with 1,000 repetitions by using R software (R version 3.5.3 binary for OS X 10.11, EI Capitan; R Core Team, 2019). The cut-off values for the pNEWS and pMEWS were determined by using Youden’s index (sensitivity + specificity −1). Using these determined cut-off points, the sensitivity, specificity and odds ratio of pNEWS and pMEWS were calculated for the prediction of admission and in-hospital mortality. A *p*-value of less than 0.05 was considered to indicate statistical significance. Calibration was assessed statistically using the Hosmer–Lemeshow C statistic. A statistically significant result suggests a lack of calibration. Sample size was calculated by events per variable (EPVs). Peduzzi et al. (1996) demonstrated that 10 EPVs were required for accurate estimation of regression coefficients in a logistic regression model. We calculated seven variables for NEWS and six variables for MEWS in our study, so we needed more than 70 events. Previous studies reported that mortality rates were 4.7–6.9% (Abbott et al., 2016, 2018), and we assumed the mortality rate to be 5.8%. Finally, we set the sample size at 1,210 cases. Data were analysed by the Statistical Package for the Social Sciences, version 16.0 (SPSS, Chicago, IL, USA).

## Results

During the study period, 2,204 elderly patients presented to the ED by ambulance. Pre-hospital data on vital signs were missing for 916 patients (41.6%), and ED data on vital signs were missing for 595 patients (27.0%). We recovered the data completely by using multiple imputation analysis.

The median age (interquartile range) of the patients was 78 (11) years, and 1,188 (53.9%) patients were male. The major diagnostic categories were 373 (16.9%) trauma cases, 492 (22.3%) cardiology cases, 304 (13.8%) neurology cases, 305 (13.8%) gastroenterology cases, 174 (7.9%) pulmonology cases, 132 (6.0%) nephrology/urology cases, 130 (5.9%) otolaryngology cases and 129 (5.9%) other cases (multi-organ failure, severe sepsis, heat stroke, etc.). The median length (interquartile range) of stay in the ED was 125 (114) min. A total of 868 (39.4%) patients were discharged from the ED, 938 (42.6%) patients were admitted to a ward, and 398 (18.1%) patients were admitted to the ICU. A total of 127 (5.8%) patients died within 28 days of presenting to the ED. Among patients with incomplete pre-hospital or ED data, more patients were discharged from the ED than were admitted to hospital; moreover, this group of patients had a lower mortality rate than did patients with complete pre-hospital and ED data (Table 3).

**Table 3:** Baseline characteristics of the study population.

	Total population (*n* = 2,204) Median (interquartile range)	Pre-hospital			Emergency department		
		Data complete group (*n* = 1,288) Median (interquartile range)	Data incomplete group (*n* = 916) Median (interquartile range)	*p*-value	Data complete group (*n* = 1,609) Median (interquartile range)	Data incomplete group (*n* = 595) Median (interquartile range)	*p*-value
Age, years	78 (11)	78 (11)	78 (10)	0.326	78 (11)	78 (10)	0.214
Sex (*n* (%))				<0.05			<0.05
Male	1,188 (53.9)	719 (55.8)	469 (51.2)		896 (55.7)	292 (49.1)	
Female	1,016 (46.1)	569 (44.2)	447 (48.8)		713 (44.3)	303 (50.9)	
Diagnostic category (*n* (%))				<0.001			<0.001
Trauma	373 (16.9)	219 (17.0)	154 (16.8)		274 (17.0)	99 (16.6)	
Cardiology	492 (22.3)	391 (30.4)	101 (11.0)		440 (27.3)	52 (8.7)	
Neurology	304 (13.8)	201 (15.6)	103 (11.2)		242 (15.0)	62 (10.4)	
Gastroenterology	305 (13.8)	148 (11.5)	157 (17.1)		206 (12.8)	99 (16.6)	
Pulmonology	174 (7.9)	128 (9.9)	46 (5.0)		149 (9.3)	25 (4.2)	
Nephrology/Urology	132 (6.0)	54 (4.2)	78 (8.5)		80 (5.0)	52 (8.7)	
Otolaryngology	130 (5.9)	15 (1.2)	115 (12.6)		37 (2.3)	93 (15.6)	
Endocrinology	36 (1.6)	19 (1.5)	17 (1.9)		24 (1.5)	12 (2.0)	
Hematology	36 (1.6)	13 (1.0)	23 (2.5)		23 (1.4)	13 (2.2)	
Dermatology	23 (1.0)	10 (0.8)	13 (1.4)		13 (0.8)	10 (1.7)	
Ophthalmology	22 (1.0)	1 (0.1)	21 (2.3)		2 (0.1)	20 (3.4)	
Gynecology	18 (0.8)	8 (0.6)	10 (1.1)		10 (0.6)	8 (1.3)	
Psychiatry	18 (0.8)	5 (0.4)	13 (1.4)		7 (0.4)	11 (1.8)	
Toxicology	7 (0.3)	7 (0.5)	0 (0)		7 (0.4)	0 (0)	
Collagen disease	5 (0.2)	3 (0.2)	2 (0.2)		4 (0.2)	1 (0.2)	
Others	129 (5.9)	66 (5.1)	63 (6.9)		89 (5.5)	38 (6.4)	
Length of stay in ED (min)	125 (114)	132 (113)	113 (114)	<0.001	133 (115)	106 (109)	<0.001
Disposition (*n* (%))				<0.001			<0.001
Discharge	868 (39.4)	359 (27.9)	510 (55.7)		469 (29.1)	399 (67.1)	
Admission to a ward	938 (42.6)	584 (45.3)	353 (38.5)		755 (46.9)	183 (30.8)	
Admission to ICU	398 (18.1)	345 (26.8)	53 (5.8)		385 (23.9)	13 (2.2)	
Death (*n* (%))	127 (5.8)	97 (7.5)	30 (3.3)	<0.001	114 (7.1)	13 (2.2)	<0.001

The median pNEWS/eNEWS and pMEWS/eMEWS of patients admitted to a ward or the ICU were significantly higher than the median pNEWS/eNEWS and pMEWS/eMEWS of patients discharged from the ED. The eNEWS was significantly higher than the pNEWS in patients who were discharged from the ED and admitted to a ward, but there was no significant difference between the eNEWS and the pNEWS in patients who were admitted to the ICU. The eMEWS was significantly higher than the pMEWS in patients who were discharged from the ED and admitted to a ward, but there was no significant difference between the eMEWS and the pMEWS in patients who were admitted to the ICU (Table 4).

**Table 4:** Comparison of parameters between the discharged group, the admitted to a ward group, and the admitted to ICU group.

	Median (interquartile range)			*p*-value	*p*-values of paired comparisons		
	Group 1 (Discharged from ED) (*n* = 868)	Group 2 (Admission to a ward) (*n* = 938)	Group 3 (Admission to ICU) (*n* = 398)		G1–G2	G2–G3	G1–G3
Age, years	78 (10.0)	79 (10.0)	77 (10.8)	<0.001	<0.05	<0.001	0.440
Sex (*n* (%))				<0.001	<0.001	<0.05	<0.001
Male	411 (47.4)	527 (56.2)	250 (62.8)				
Female	457 (52.6)	411 (43.8)	148 (37.2)				
Category (*n* (%))				<0.01	0.716	<0.01	<0.01
Trauma	158 (18.2)	177 (18.9)	48 (12.1)				
Non-trauma	710 (81.8)	761 (81.1)	350 (87.9)				
Length of stay in ED (min)	110 (94)	146 (120)	113 (120)	<0.001	<0.001	<0.001	0.418
(Pre-hospital)							
Respiratory rate (bpm)	20 (6.0)	20 (7.0)	24 (9.0)	<0.001	<0.001	<0.001	<0.001
Oxygen saturation (%)	98 (5.0)	96 (6.0)	97 (6.0)	<0.001	<0.001	1.00	<0.001
Inhaled oxygen				<0.001	<0.001	<0.001	<0.001
Yes	123 (14.2)	256 (27.3)	253 (63.6)				
No	745 (85.8)	682 (72.7)	145 (36.4)				
Temperature (°C)	36.4 (1.3)	36.7 (1.4)	36.4 (0.9)	<0.001	<0.001	<0.001	0.628
Systolic blood pressure (mmHg)	152 (45.0)	144 (47.0)	140 (57.0)	<0.001	<0.001	<0.05	<0.001
Pulse rate (bpm)	83 (32.0)	87 (35.0)	89 (37.0)	<0.001	<0.001	0.751	<0.001
AVPU (*n* (%))				<0.001	<0.01	<0.001	<0.001
Alert	640 (73.7)	716 (76.3)	282 (70.9)				
Voice	76 (8.8)	88 (9.4)	70 (17.6)				
Pain	113 (13.0)	77 (8.2)	26 (6.5)				
Unresponsive	39 (4.5)	57 (6.1)	20 (5.0)				
pNEWS	4 (5.0)*	5 (6.0)*	5 (6.0)	<0.001	<0.01	<0.01	<0.001
pMEWS	2 (3.0)*	3 (4.0)*	3 (3.0)	<0.001	<0.01	0.194	<0.001
(Emergency Department)							
Respiratory rate (bpm)	18 (8.0)	19 (8.0)	20 (9.0)	<0.001	<0.01	<0.001	<0.001
Oxygen saturation (%)	97 (4.0)	98 (4.0)	98 (4.0)	<0.001	0.254	<0.001	<0.001
Inhaled oxygen				<0.001	<0.001	<0.001	<0.001
Yes	174 (20.0)	305 (37.3)	263 (66.1)				
No	694 (80.0)	588 (62.7)	135 (33.9)				
Temperature (°C)	36.5 (1.0)	36.8 (1.2)	36.4 (1.0)	<0.001	<0.001	<0.001	0.233
Systolic blood pressure (mmHg)	149 (40.0)	142 (41.0)	139 (52.0)	<0.001	<0.001	0.104	<0.001
Pulse rate (bpm)	81 (26.0)	84 (27.0)	89 (38.0)	<0.001	<0.001	<0.01	<0.001
AVPU (*n* (%))				<0.001	<0.001	<0.001	<0.001
Alert	803 (92.5)	785 (83.7)	279 (70.1)				
Voice	44 (5.1)	101 (10.8)	75 (18.8)				
Pain	10 (1.2)	32 (3.4)	23 (5.8)				
Unresponsive	11 (1.3)	20 (2.1)	21 (5.3)				
eNEWS	3 (4.0)*	3 (4.0)*	6 (6.0)	<0.001	<0.001	<0.001	<0.001
eMEWS	2 (2.0)*	2 (3.0)*	3 (4.0)	<0.001	<0.001	<0.001	<0.001

**Notes:**

Data are presented as the median (interquartile range) for continuous variables and the number (%) for categorical variables. pNEWS, pre-hospital National Early Warning Score; pMEWS, pre-hospital Modified Early Warning Score; eNEWS, emergency department National Early Warning Score; eMEWS, emergency department Modified Early Warning Score; bpm, beats or breaths per minute; G1, Group 1; G2, Group 2, G3; Group 3.

* The *p*-value is less than 0.05 between pre-hospital and emergency department.

The median pNEWS/eNEWS and pMEWS/eMEWS were significantly higher in non-survivors than in survivors. The eNEWS and eMEWS were significantly lower than the pNEWS and pMEWS in survivors. The proportion of patients who had oxygen supplementation or bad consciousness in either the pre-hospital setting or the ED was significantly higher in non-survivors than in survivors (Table 5).

**Table 5:** Comparison of parameters between the survivors and non-survivors.

	Median (interquartile range)		*p*-value
	Group 1 (Survivors) (*n* = 2,077)	Group 2 (Non-survivors) (*n* = 127)	
Age, years	78 (11.0)	80 (9.5)	0.068
Sex (*n* (%))			0.053
Male	1,109 (53.4)	79 (62.2)	
Female	968 (46.6)	48 (37.8)	
Category (*n* (%))			<0.001
Trauma	375 (18.1)	8 (6.3)	
Non-trauma	1,702 (81.9)	119 (93.7)	
Length of stay in ED (min)	122 (112)	159 (112)	<0.01
(Pre-hospital)			
Respiratory rate (bpm)	20.0 (7.0)	24.0 (10.0)	<0.001
Oxygen saturation (%)	97.0 (4.0)	95.0 (11.0)	<0.001
Inhaled oxygen			<0.01
Yes	479 (23.1)	45 (35.4)	
No	1,598 (76.9)	82 (64.6)	
Temperature (°C)	36.5 (1.2)	36.6 (1.3)	0.083
Systolic blood pressure (mmHg)	147 (49.0)	136.0 (46.0)	<0.01
Pulse rate (bpm)	85 (32.0)	96.0 (35.0)	<0.01
AVPU (*n* (%))			<0.001
Alert	1,579 (76.0)	59 (46.5)	
Voice	201 (9.7)	33 (26.0)	
Pain	195 (9.4)	21 (16.5)	
Unresponsive	102 (4.9)	14 (11.0)	
pNEWS	4 (5.0)*	7 (5.0)	<0.001
pMEWS	3 (3.0)*	4 (2.0)	<0.001
(Emergency department)			
Respiratory rate (bpm)	19.0 (7.0)	22.0 (10.0)	<0.001
Oxygen saturation (%)	97.0 (4.0)	97.0 (5.0)	<0.001
Inhaled oxygen			<0.001
Yes	689 (33.2)	98 (77.2)	
No	1,388 (66.8)	29 (22.8)	
Temperature (°C)	36.6 (1.1)	36.7 (1.4)	0.196
Systolic blood pressure (mmHg)	145 (40.0)	128.0 (53.0)	<0.001
Pulse rate (bpm)	83 (28.0)	95.0 (30.0)	<0.001
AVPU (*n* (%))			<0.001
Alert	1,809 (87.1)	58 (45.7)	
Voice	186 (9.0)	34 (26.8)	
Pain	51 (2.5)	14 (11.0)	
Unresponsive	31 (1.5)	21 (16.5)	
eNEWS	3 (5.0)*	8 (5.0)	<0.001
eMEWS	2 (3.0)*	4 (3.0)	<0.001

**Notes:**

Data are presented as the median (interquartile range) for continuous variables and the number (%) for categorical variables. pNEWS, pre-hospital National Early Warning Score; pMEWS, pre-hospital Modified Early Warning Score; eNEWS, emergency department National Early Warning Score; eMEWS, emergency department Modified Early Warning Score; bpm, beats or breaths per minute.

* The *p*-value is less than 0.05 between pre-hospital and emergency department.

**Figure 1:**
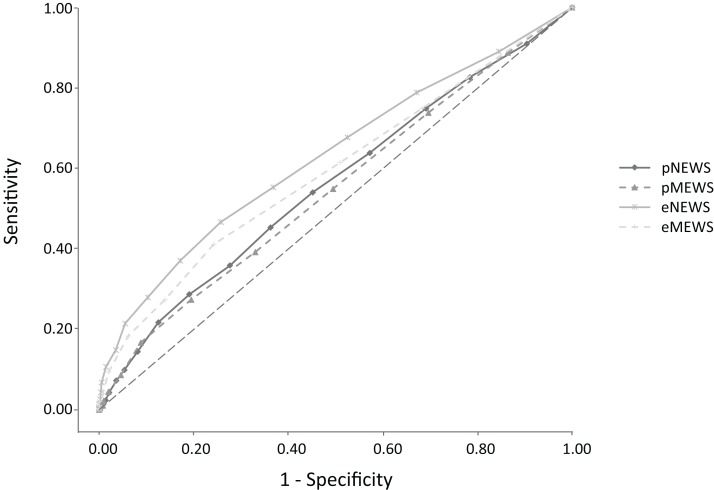
Receiver operator characteristic (ROC) curves for admission comparing the Early Warning Scores in the pre-hospital and in the emergency department.

**Figure 2:**
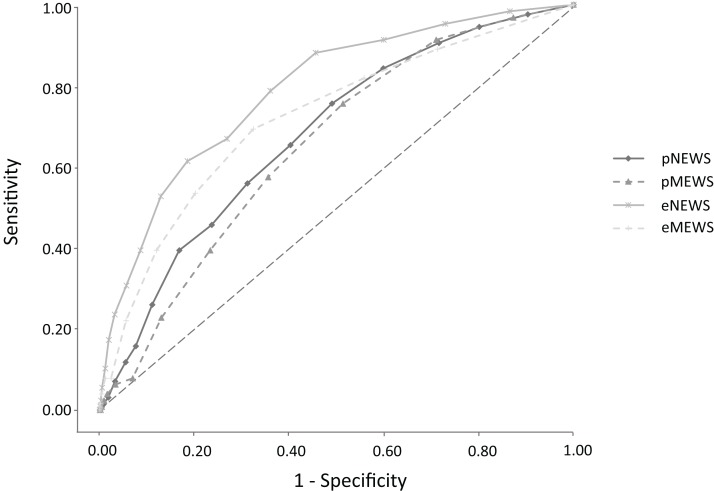
Receiver operator characteristic (ROC) curves for in-hospital mortality comparing the Early Warning Scores in the pre-hospital and in the emergency department.

The AUC for predicting admission was 0.559 (95% CI [0.536–0.583], *p* < 0.001) for the pNEWS and 0.547 (95% CI [0.525–0.572], *p* < 0.001) for the pMEWS. There was no significant difference between the AUC of the pNEWS and the pMEWS for predicting admission (*p* = 0.102). The cut-off values for admission were 5 for the pNEWS and 3 for the pMEWS. A pNEWS of 5 or more had a sensitivity of 54.0%, a specificity of 54.8% and an odds ratio of 1.43 for predicting admission. A pMEWS of 3 or more had a sensitivity of 54.9%, a specificity of 50.6% and an odds ratio of 1.25 for predicting admission (Fig. 1). The AUC for predicting in-hospital mortality was 0.678 (95% CI [0.633–0.720], *p* < 0.001) for the pNEWS and 0.652 (95% CI [0.609–0.695], *p* < 0.001) for the pMEWS. There was no significant difference between the AUC of the pNEWS and the pMEWS for predicting in-hospital mortality (*p* = 0.081). The cut-off values for in-hospital mortality were 6 for the pNEWS and 4 for the pMEWS. A pNEWS value of 6 or more had a sensitivity of 65.4%, a specificity of 59.7% and an odds ratio of 2.79 for predicting in-hospital mortality. A pMEWS value of 4 or more had a sensitivity of 57.5%, a specificity of 64.5% and an odds ratio of 2.45 for predicting in-hospital mortality (Fig. 2). The AUC for predicting admission was 0.628 (95% CI [0.605–0.652], *p* < 0.001) for the eNEWS and 0.591 (95% CI [0.569–0.616], *p* < 0.001) for the eMEWS. The cut-off values for admission were 4 for the eNEWS and 3 for the eMEWS. An eNEWS of 4 or more had a sensitivity of 55.3%, a specificity of 63.1% and an odds ratio of 2:12 for predicting admission. An eMEWS of 3 or more had a sensitivity of 41.2%, a specificity of 75.7% and an odds ratio of 2.18 for predicting admission (Fig. 1). The AUC for predicting in-hospital mortality was 0.789 (95% CI [0.747–0.829, *p* < 0.001) for the eNEWS and 0.720 (95% CI [0.671–0.765], *p* < 0.001) for the eMEWS. The cut-off values for in-hospital mortality were 5 for the eNEWS and 3 for the eMEWS. An eNEWS of 5 or more had a sensitivity of 78.7%, a specificity of 64.0% and an odds ratio of 6:58 for predicting in-hospital mortality. An eMEWS value of 3 or more had a sensitivity of 69.3%, a specificity of 67.6% and an odds ratio of 4:71 for predicting in-hospital mortality (Fig. 2).

For admission and in-hospital mortality, the AUC of the eNEWS was significantly greater than that of the pNEWS (*p* < 0.001, *p* < 0.001), and the AUC of the eMEWS was significantly greater than that of the pMEWS (*p* < 0.01, *p* < 0.05).

Except for the eMEWS at admission, all scores were well calibrated for both admission and in-hospital mortality (Table 6).

**Table 6:** AUC and Hosmer–Lemeshow of Fit Test for the prediction of the need for admission and in-hospital mortality.

Score	AUC (95% CI)	Hosmer–Lemeshow C statistic (Chi-Square)
Admission		
pNEWS	0.559 [0.536–0.583]	10.287
pMEWS	0.547 [0.525–0.572]	9.868
eNEWS	0.628 [0.605–0.652]	14.000
eMEWS	0.591 [0.569–0.616]	20.859*
In-hospital mortality		
pNEWS	0.678 [0.633–0.720]	3.555
pMEWS	0.652 [0.609–0.695]	8.960
eNEWS	0.789 [0.747–0.829]	11.443
eMEWS	0.720 [0.671–0.765]	2.864

**Note:**

* *p* < 0.05.

## Discussion

Several studies have evaluated the effectiveness of risk-scoring systems for predicting admission to critical care units from wards or in-hospital mortality in the last decade, but many of them focused on in-hospital management (Subbe et al., 2001; Cei, Bartolomei & Mumoli, 2009; Groot et al., 2017). Few studies set in the ED have shown the predictive value of the NEWS and MEWS for hospitalisation or in-hospital mortality (Lee et al., 2018). Moreover, few studies from the pre-hospital setting have shown the predictive value of the NEWS for escalation to the critical care unit within 48 h after hospital admission or death (Abbott et al., 2018; Hoikka, Silfvast & Ala-Kokko, 2018).

A study by Groot et al. (2017) demonstrated the low prognostic performance of the NEWS for risk stratification in elderly patients with sepsis, but there has been no other study devoted to elderly patients. Thus, the performance of risk-scoring systems for elderly patients in the pre-hospital setting is still controversial.

Although previous studies that estimated the prognostic value of risk-scoring systems did not include patients with trauma, we included such patients in our study because elderly patients frequently have not only endogenous diseases but also traumatic problems in the clinical setting.

In a study by Sbiti-Rohr et al. (2016), the AUC of the NEWS for ICU admission was 0.73. No study has calculated the AUC of the NEWS for total admissions, which would include patients who were admitted to the ward. In the studies by Subbe et al. (2001) and Bulut et al. (2014), the AUC of the MEWS for admission to the ward or the ICU was 0.62–0.568. Although several studies of the AUC of the NEWS and the MEWS for admission have been carried out, none of them calculated the AUC of the NEWS or the MEWS for admission in elderly patients.

Our study examined the ability of the pNEWS/eNEWS and the pMEWS/eMEWS to predict admission in elderly patients, but none of these scores had excellent (AUC > 0.90) or good (AUC > 0.80) ability to predict admission.

We found that the pNEWS/eNEWS and the pMEWS/eMEWS were poorly effective for predicting admission in elderly patients; the AUCs for admission were 0.559/0.628 and 0.547/0.591. There was no significant difference between the AUC of the pNEWS and the pMEWS for predicting admission, whereas the AUC of the eNEWS was significantly greater than that of the eMEWS.

In studies by Smith et al. (2013) and Kovacs et al. (2016), the predictive value of the NEWS for in-hospital mortality was very high, with an AUC of 0.894–0.902. In studies by Bulut et al. (2014) and Smith et al. (2013), the predictive value of the MEWS for in-hospital mortality was also high, yielding an AUC of 0.630–0.865. Although several studies of the NEWS and the MEWS for predicting in-hospital mortality were carried out, none of them calculated the AUC of the NEWS or the MEWS for in-hospital mortality in elderly patients. Only a few studies calculated the predictive value of the pNEWS. In the study by Pirneskoski et al. (2019), the predictive value of the NEWS for in-hospital mortality within 1 day was high, with an AUC of 0.840, but the study was not based on the elderly population.

We found that the pNEWS and the pMEWS had low effectiveness, and the eNEWS and the eMEWS had moderate effectiveness for predicting in-hospital mortality in elderly patients. Our study also found that there was no significant difference between the AUC of the pNEWS and the pMEWS for predicting in-hospital mortality, whereas the AUC of the eNEWS was significantly greater than that of the eMEWS.

In previous studies by Abbott et al. (2016, 2018), the mortality rate was 4.7% (15 of 322 patients) to 6.9% (13 of 189 patients). In our study, the in-hospital mortality rate within 28 days after admission was 5.8% (127 of 2,204 patients), which is similar to that in the previous study. The EPVs were 18.1, and we secured the number of event cases necessary for accurate estimation.

The NEWS and MEWS have been introduced to the ED to predict patients’ prognosis in the United Kingdom. However, because there is not much evidence for the value of these risk scores in the pre-hospital setting for predicting admission and in-hospital mortality, these risk scores have not been introduced in the pre-hospital setting (Abbott et al., 2018). In the past two decades, EMS crews in Japan have used a severity and urgency criterion that is based on physiological evaluation, anatomical evaluation, symptoms and mechanism of injury. This criterion is not based on international triage systems, such as the Canadian Triage and Acuity Scale, and it is not updated regularly (Foundation for Ambulance Service Development, 2004).

No study has evaluated the usefulness of pre-hospital risk-scoring systems for predicting patients’ prognosis in Japan. Because it is very difficult to predict the severity of illness in elderly patients, we need to accumulate more evidence of the value of pre-hospital clinical risk scores, in order to more clearly anticipate the patient’s prognosis and to perform appropriate triage.

Our study has shown that the pNEWS and the pMEWS have low utility as predictors of patient admission and in-hospital mortality. Therefore, it is difficult to use these scores as criteria for judging whether hospitalisation is necessary in the pre-hospital setting or for judging whether the EMS crew should transport the patient to a high-level emergency institution as rapidly as possible.

The AUCs for admission and in-hospital mortality of elderly patients in our study were lower than those of previous studies due to the inclusion of patients with trauma, the large amount of missing data and the distinctive physical signs, such as dementia.

This study has several limitations. First, it was a retrospective, single-centre study. Second, discharged patients were not followed up for readmission to the ED and out-of-hospital mortality. Third, the proportion of cases with missing data was relatively high at about 40%. These limitations may reduce the generalizability of the results. Further multicentre studies are needed for external validation and to remove selection bias. Several other pre-hospital risk-scoring systems need to be studied to determine which are good predictors of patient admission and in-hospital mortality.

## Conclusions

Our single-centre study has demonstrated the low utility of the pNEWS and the pMEWS as predictors of admission and in-hospital mortality in elderly patients, whereas the eNEWS and the eMEWS predicted admission and in-hospital mortality more accurately. Evidence from multicentre studies is needed before introducing pre-hospital versions of risk-scoring systems.

## Supplemental Information

10.7717/peerj.6947/supp-1 Supplemental Information 1 The raw data of this study. In the data set Emergency Department, we collected the first vital signs just after arrival at the ED. In the data set pre-hospital, we collected the vital signs which are gathered by EMS crews. The non-colored cells are the patients whose all vital information are collected, while the yellow cells are the patients whose vital signs are not collected totally.

10.7717/peerj.6947/supp-2 Supplemental Information 2 The original study protocol which is approved by the ethics committee of Jikei university school of medicine and Chiba university (Japanese).

10.7717/peerj.6947/supp-3 Supplemental Information 3 The study protocol which is approved by the committee of Jikei university school of medicine and Chiba university (English).

## Additional Information and Declarations

### Data Availability

The following information was supplied regarding data availability: The raw measurements are available in the Supplemental File.

## References

[ref-1] Abbott TEF, Cron N, Vaid N, Ip D, Torrance HDT, Emmanuel J (2018). Pre-hospital national early warning score (NEWS) is associated with in-hospital mortality and critical care unit admission: a cohort study. Annals of Medicine and Surgery.

[ref-2] Abbott TEF, Torrance HDT, Cron N, Vaid N, Emmanuel J (2016). A single-centre cohort study of National Early Warning Score (NEWS) and near patient testing in acute medical admissions. European Journal of Internal Medicine.

[ref-3] Bulut M, Cebicci H, Sigirli D, Sak A, Durmus O, Top AA, Kaya S, Uz K (2014). The comparison of modified early warning score with rapid emergency medicine score: a prospective multicentre observational cohort study on medical and surgical patients presenting to emergency department. Emergency Medicine Journal.

[ref-4] Burch VC, Tarr G, Morroni C (2008). Modified early warning score predicts the need for hospital admission and in hospital mortality. Emergency Medicine Journal.

[ref-5] Cei M, Bartolomei C, Mumoli N (2009). In-hospital mortality and morbidity of elderly medical patients can be predicted at admission by the Modified Early Warning Score: a prospective study. International Journal of Clinical Practice.

[ref-6] Foundation for Ambulance Service Development (2004). The report of committee for preparing criteria for severity/urgency criteria for emergency transport. http://www.fasd.or.jp/tyousa/hanso01.pdf.

[ref-7] Groot B, Stolwijk F, Warmerdam M, Lucke JA, Singh GK, Abbas M, Mooijaart SP, Ansems A, Esteve Cuevas L, Rijpsma D (2017). The most commonly used disease severity scores are inappropriate for risk stratification of older emergency department sepsis patients: an observational multi-centre study. Scandinavian Journal of Trauma, Resuscitation and Emergency Medicine.

[ref-8] Hoikka M, Silfvast T, Ala-Kokko TI (2018). Does the prehospital National Early Warning Score predict the short-term mortality of unselected emergency patients?. Scandinavian Journal of Trauma, Resuscitation and Emergency Medicine.

[ref-9] Kovacs C, Jarvis SW, Prytherch DR, Meredith P, Schmidt PE, Briggs JS, Smith GB (2016). Comparison of the National Early Earning Score in non-elective medical and surgical patients. British Journal of Surgery.

[ref-10] Lee YS, Choi JW, Park YH, Chung C, Park DI, Lee JE, Lee HS, Moon JY (2018). Evaluation of the efficacy of the National Early Warning Score in predicting in-hospital mortality via the risk stratification. Journal of Critical Care.

[ref-11] Lee YS, Lee JW, Lee J, Min NE, Park JE, Jung JW, Park DI, Kim KD, Ahn HJ, Choi JW, Park YH, Ryu S, Jeong WJ, Moon JY (2015). The usefulness of modified national early warning score with the age level in critically ill medical patients. Intensive Care Medicine Experimental.

[ref-12] Mitusnaga T, Hujita M, Hasegawa I, Otani K, Okuno K, Ohtaki Y, Seki Y, Mashiko K, Takeda S (2018). Abbreviated National Early Warning Score predicts the need for hospital admission and in-hospital mortality in elderly patients. Emergency Care Journal.

[ref-13] Peduzzi P, Concato J, Kemper E, Holford TR, Feinstein AR (1996). A simulation study of the number of events per variable in logistic regression analysis. Journal of Clinical Epidemiology.

[ref-14] Pirneskoski J, Kuisma M, Olkkola KT, Nurmi J (2019). Prehospital National Early Warning Score predicts early mortality. Acta Anaesthesiologica Scandinavica.

[ref-21] R Core Team (2019). R: A language and environment for statistical computing.

[ref-15] Royal College of Physicians London (2012). National Early Warning Score (NEWS): Standardising the assessment of acute-illness severity in the NHS.

[ref-16] Sbiti-Rohr D, Kutz A, Christ-Crain M, Thomann R, Zimmerli W, Hoess C, Henzen C, Mueller B, Schuetz P, ProHOSP Study Group (2016). The National Early Warning Score (NEWS) for outcome prediction in emergency department patients with community-acquired pneumonia: results from a 6-year prospective cohort study. BMJ Open.

[ref-17] Smith GB, Prytherch DR, Meredith P, Schmidt PE, Featherstone PI (2013). The ability of the National Early Warning Score (NEWS) to discriminate patients at risk of early cardiac arrest, unanticipated intensive care unit admission, and death. Resuscitation.

[ref-18] Subbe CP, Kruger M, Rutherford P, Gemmel L (2001). Validation of a modified Early Warning Score in medical admissions. QJM—An International Journal of Medicine.

[ref-19] Tanigawa K, Tanaka K (2006). Emergency medical service systems in Japan: Past. Present, and future. Resuscitation.

[ref-20] World Health Statistics (2017). Monitoring health for the SDGs. https://apps.who.int/iris/bitstream/handle/10665/255336/9789241565486-eng.pdf;jsessionid=BF0AA99B2DC5D5B1CC016658EC82D469?sequence=1.

