# Supplementary material for: Comparison of the National Early Warning Score (NEWS) and the Modified Early Warning Score (MEWS) for predicting admission and in-hospital mortality in elderly patients in the pre-hospital setting and in the emergency department
Source: PeerJ. 2019 May 16;7:e6947. doi: 10.7717/peerj.6947 (PMC6526008; doi:10.7717/peerj.6947)
Supplement: Supplemental Information 2 [file peerj-07-6947-s002.pdf]

研究実施計画書

第１版　2018 年５月 10 日

第７版　2019 年１月１８日

課題名「救急搬送された高齢患者の予後予測スコアは、死亡率・入院率や救急部滞在時間と関連性があるか」

１．研究の組織・実施体制（研究機関の名称及び研究者等の氏名・役割を含む。）

〈研究代表者〉

光永敏哉　東京慈恵会医科大学　救急医学講座　助教　研究計画の立案・データ管理者責任者

〈研究分担者〉

武田　聡　東京慈恵会医科大学　救急医学講座　教授　研究計画アドバイザー

卯津羅　雅彦　東京慈恵会医科大学　救急医学講座　教授　研究計画アドバイザー

大谷　圭　東京慈恵会医科大学　救急医学講座　准教授　研究計画アドバイザー・データ収集責任者

長谷川　意純　東京慈恵会医科大学　救急医学講座　助教　データ収集担当者

〈共同研究者〉

関根　章博　千葉大学　予防医学センター　教授　データ解析担当者・研究計画アドバイザー

２．研究の目的及び意義

＜目的＞　近年増加傾向にある高齢救急患者の Rapid Acute Physiology Score (RAPS)/ Rapid Emergency Medicine Score (REMS)/ National Early Warning Score (NEWS)/ Modified Early Warning Score (MEWS) と死亡率・入院率や救急部滞在時間との関連性を明らかにする。

＜意義＞

近年，多くの医療機関における救急外来の過剰な混雑状態が問題となっている。中でも，救急現場での軽症患者数の増加により，重症救急患者への対応の遅れや患者の訴えの見逃しだけでなく，救急医療従事者の過重労働など問題が山積している。救急隊による年間の救急搬送件数は1990年に約270万人であったのが，2000年に約400万人，2012年には約520万人に増加しており，これは国民の24人に1人が救急隊によって搬送されたことになる。搬送患者の中でも，特に軽症者の増加が著しい。また，平成24年度119番通報入電から病院等に収容するまでの平均時間は全国で38.7分であるが，東京都では平成24年度54.9分と47都道府県中最下位となっており，救急外来受診の適正化と速やかな救急要請の受け入れは急務である。
搬送される患者のうち、65 歳以上の高齢者の数がこの 10 年間で激増している。高齢患者では、主訴の曖昧さから時に診察が困難であり、検査数の増加や複数の診療科にコンサルトするために救急部滞在時間が長くなってしまう。救急外来における高齢患者のトリアージをいかに素早く行うかが、救急部の円滑な運営にとっても必要不可欠である。
Rapid Acute Physiology Score (RAPS)/ Rapid Emergency Medicine Score (REMS)/ National Early Warning Score (NEWS)/ Modified Early Warning Score (MEWS)はベッドサイドで患者の急変予測を行うツールとして、世界各地で導入された。これらスコアの導入によって院内の死亡率が低下したり、緊急の ICU 入室率を低下させることができた。
我々は、これらのスコアを用いて、救急外来に搬送された高齢患者の入院や救急部滞在時間との関連性を解析し、高齢救急患者のトリアージツールとしての有用性を検討する。

３．研究の方法

・**研究デザイン：**Retrospective Observational Study
・**研究対象者：**2017 年４月１日から 2018 年３月 31 日までの期間に東京慈恵会医科大学附属第三病院救急部と東京慈恵会医科大学附属柏病院救命救急センターを受診した患者のうち以下の症例を対象とする。

【Inclusion criteria:】

1）救急搬送された 65 歳以上のすべての患者

・**研究方法：**

●本研究は千葉大学との共同研究であり、本学は観察および検査等で得られた情報を千葉大学と共有し解析を行う。

●Rapid Acute Physiology Score (RAPS)

	0	+1	+2	+3	+4
脈拍数	70-109		55-69<br>110-139	40-54<br>140-179	39 以下<br>180 以上
MAP	70-109		50-69<br>110-129	130-159	49 以下<br>160 以上
呼吸数	12-24	10-11	6-9	35-49	5 以下

		25-34			50 以上
GCS	14 以上	11-13	8-10	5-7	4 以下

●Rapid Emergency Medicine Score (REMS)

	0	+1	+2	+3	+4	+5	+6
年齢	45 以下		45-54	55-64		65-74	74 以上
脈拍数	70-109		55-69<br>110-139	40-54<br>140-179	39 以下<br>179 以上		
MAP	70-109		50-69<br>110-129	130-159	49 以下<br>159 以上		
呼吸数	12-24	10-11<br>25-34	6-9	35-49	5 以下<br>49 以上		
GCS	14 or 15	11-13	8-10	5-7	3 or 4		
SpO2	89 以上	86-89		75-85	75 以下		

●National Early Warning Score (NEWS)

	3	2	1	0	1	2	3
呼吸数	8 以下		9-11	12-20		21-24	25 以上
SpO2	91 以下	92-93	94-95	96 以上			
酸素投与		Yes		No			
体温	35 以下		35.1-36.0	36.1-38.0	38.1-39.0	39.1 以上	
収縮期血圧	90 以下	91-100	101-110	111-219			220 以上
脈拍数	40 以下		41-50	51-90	91-110	111-130	131 以上
意識覚醒				A			V,P, or U

●Modified Early Warning Score (MEWS)

Points	3	2	1	0	1	2	3
体温<br>°C		35 以下		35.1-38.4		38.5 以上	
脈拍数<br>bpm		39 以下	40-50	51-100	101-110	111-129	130 以上
収縮期血圧<br>mmHg	70 以下	71-80	81-100	101-199		200 以上	
呼吸数		8 以下	9	10-18	19-20	21-29	30 以上
AVPU				A	V	P	U

●電子カルテから、救急外来ならびに救急隊が病院前で記載した上記スコアに必要な①意識状態、②脈拍数、③呼吸数、④収縮期血圧 or MAP、⑤体温、⑥SpO2、⑦酸素投与、⑧年齢に関する情報を抽出する。

●患者情報として、①来院月、②来院時刻、③年齢、④性別、⑤診断カテゴリー（総合内科・呼吸器内科・循環器内科・消化器肝臓内科・神経内科・血液腫瘍内科・糖尿病代謝内分泌内科・腎臓高血圧内科・膠原病内科・外科・整形外科・脳神経外科・形成外科・泌尿器科・耳鼻咽喉科・その他）、⑥既往歴、⑦認知症の有無・程度、⑧ADL、⑨生活場所（他医療機関・福祉施設・自宅）、⑩転帰（帰宅・ICU 入院・一般床入院・28 日死亡率）、⑩採血項目（CRP・アルブミン値・血液ガス）を収集する。

・**主要評価項目（Primary endpoint）：**

●上記各スコアの点数と入院・滞在時間に相関関係があるか。

・**実施場所：**

① 東京慈恵会医科大学附属第三病院救急部

② 東京慈恵会医科大学附属柏病院救命救急センター

・**統計学的解析：**本試験の評価項目として設定した種々の観察項目のデータのカテゴリーに従い，適切な統計解析を、統計ソフトを用いて比較検討する．統計解析は救急医学講座医局内（本院）または、千葉大学予防医学センターオミクス応用解析研究室内にて行う。著名化された情報は、パスワード付き USB メモリーに保管し、研究代表者が千葉大学へ持参する。

4． 目標症例数及び予定期間

＜目標症例数＞

参加施設：2 施設

目標対象者：2500 例

＜症例数の設定根拠＞

昨年度東京慈恵会医科大学附属第三病院救急部の救急車収容台数は約 3,500 台で東京慈恵会医科大学附属柏病院救命救急センターの救急車収容台数は約 5,000 台であった。H29 年度総務省消防庁のまとめでは、全救急搬送患者のうち 57.2%が 65 歳以上であった。従って、予想される 65 歳以上の患者数は 8,500(台)×1(年)×0.5＝4,250 例。データ欠損例などが除外されると予想すると，4,250×0.6=2,550 であり，目標症例数は 2,500 例と設定した。

＜研究期間＞

倫理委員会承認日より2020年3月31日迄

調査対象期間：2017年4月1日～2018年3月31日

5．研究対象者の選定方針

<登録基準>　2017 年 4 月 1 日～2018 年 3 月 31 日に東京慈恵会医科大学附属第三病院救急部ならびに東京慈恵会医科大学附属柏病院救命救急センターに救急車で搬送された 65 歳以上のすべての患者。
<除外基準>　なし

6．研究の科学的合理性の根拠

① Rapid Acute Physiology Score (RAPS)/ Rapid Emergency Medicine Score (REMS)/ National Early Warning Score (NEWS)/ Modified Early Warning Score (MEWS)はベッドサイドで患者の急変予測を行うツールとして、世界各地で導入され、これまでにアメリカにおいて Modified Early Warning Score と入院との関連性について比較した研究が報告されているが、高齢化が進む本邦における報告はない。本研究では、複数の Score について高齢患者の入院や救急部滞在時間との関連性について解析する。上記スコアが入院予測スコアとして有用であれば、今後救急外来における高齢患者の方針決定までの時間の短縮や救急外来混雑の緩和につながる可能性がある。

7．インフォームド・コンセントを受ける手続等

<インフォームド・コンセントの方法>

本研究は，人を対象とする医学系研究に関する倫理指針の規定により，研究対象者からインフォームド・コンセントを受けることを必ずしも要しないと判断されるが，当該臨床研究の目的を含む研究の実施についての情報公開を行う.

本研究では倫理委員会で承認の得られた文書を救急外来にポスター掲示すると同時にホームページ上に公開することで情報公開を行うこととし，研究が実施または継続されることについて研究対象者が拒否できる機会を保障する.

なお，情報公開文書には以下の事項を含めることとする.

1.研究概要（名称・目的・研究期間など）

2.研究に利用する情報の項目

3.他の機関へ情報等を提供する方法

4.研究計画書および研究の方法に関する資料を入手または閲覧できる旨、並びにその入手・閲覧方法

5.情報利用を拒否できる旨

6.研究代表者の氏名

<試料・情報の授受に関する記録の作成及び保管について>

本研究は，共同研究機関と情報の授受があるため，情報の提供元と提供先で別添2「資料・情報の授受に関する記録の作成および保管について」のとおりとする．

8． 個人情報等の取扱い（匿名化する場合にはその方法を含む）

<個人情報等の取扱い>

本研究に係る研究対象者の個人情報は，「学校法人慈恵大学　個人情報保護に関する規定」，「個人情報の取得・利用ならびに第三者提供に関する細則」，「医学系研究（人を対象とする医学系研究に関する倫理指針およびヒトゲノム・遺伝子解析研究に関する倫理指針に基づく研究）における個人情報保護のための安全対策管理措置手順書」および「人を対象とする医学系研究に関する倫理指針」を遵守して取り扱う．解析結果は，研究対象者にプライバシー上の不利益が生じないよう，適切に匿名化されていることを確認した上で公表する．研究代表者は、試料・情報の取得後、速やかに個人情報分担管理者に渡し匿名化を依頼する。取り扱う匿名化方法・対応表の管理方法、安全管理体制の詳細は、別添１の通りとする。本研究に係る研究対象者の特定の個人を識別できる情報は，他の研究機関と共有しない．

<個人情報保護相談窓口>

附属第三病院：03-3480-1151　内線：3710

附属柏病院：04-7164-1111　内線：2183

対応時間：午前９時～午後５時/休診日を除く

9． 予測されるリスク及び利益

<有害事象・副作用>

なし

<リスクを最小化される対策>

なし

<利益>

本研究への参加することによる研究対象者に直接の利益は生じない．研究成果により将来の救急医療の進歩に貢献できる可能性がある．

１０．　試料・情報の保管及び廃棄の方法

本研究に係る研究対象者の情報は，人体から取得された情報等の保管に関する手順書を尊守して取り扱う．

1）保管する情報：申請書類の控え，学長からの通知文書，情報の提供に関する記録，対応表，覚書

2）保管方法：別添1　個人情報の取り扱いに記載の通り．

3）破棄方法：別添1　個人情報の取り扱いに記載の通り．

4）保管期間：別添1　個人情報の取り扱いに記載の通り．

5）本研究は、他機関と試料・情報の授受があるため、試料・情報の提供元と提供先との間で別添２の「試料・情報の授受にかんする記録の作成及び保管について」の通りとする。

１１．　研究機関の長への報告内容及び方法

1）研究代表者は、研究を実施（研究計画書を変更して実施する場合を含む。以下同じ。）しようとするときは、あらかじめ研究計画書を作成し、学長及び第三病院病院長・柏病院病院長の許可を受ける。

2）研究代表者は、研究の倫理的妥当性若しくは科学的合理性を損なう事実若しくは情報又は損なうおそれのある情報であって研究の継続に影響を与えると考えられるものを得た場合には、遅滞なく、学長及び第三病院病院長・柏病院病院長に対して報告し、必要に応じて、研究を停止し、若しくは中止し、又は研究計画書を変更する。

3）研究代表者は、研究の実施の適正性若しくは研究結果の信頼を損なう事実若しくは情報又は損なうおそれのある情報を得た場合には、速やかに学長及び第三病院病院長・柏病院病院長に報告し、必要に応じて、研究を停止し、若しくは中止し、又は研究計画書を変更する。

4）研究代表者は、年に1回、研究の進捗状況及び研究の実施に伴う有害事象の発生状況、人体から取得された試料及び情報等の保管・管理状況について学長に報告する。

5）研究代表者は、研究を終了（中止の場合を含む。以下同じ。）したときは、学長及び第三病院病院長・柏病院病院長に必要な事項について報告する。また、侵襲（軽微な侵襲を除く）を伴う研究であって介入を行うものについて、結果の最終の公表を行ったときは、遅滞なく研究機関の長へ報告する。

１２．　研究の資金源と利益相反

<研究の資金源>

この研究に関する経費は，研究代表者が所属する講座・研究室の研究費で賄われる．

<利益相反および個人の収益等>

この研究は，特定の企業・団体との関与はない．研究の実施に当たり，研究の透明性および公平性の確保に努める．なお，研究者は本学の利益相反管理規定を尊守し、利益相反管理委員会への手続きを行っている．

１３．　研究に関する情報公開の方法

介入を伴う研究ではないため，公開データベースへの登録は行わない.

研究結果は、海外学会や英文誌へ発表する．

１４．　研究対象者等及びその関係者からの相談等への対応

担当者：光永敏哉

電話番号：03-3433-1111（内線：3115）

１５．　代諾者等からインフォームド・コンセントを受ける手続

レトロスペクティブ解析のため該当せず.

<インフォームド・コンセントの手続き>　なし

<代諾者の選定方針>　なし

１６．　インフォームド・アセントの手続

レトロスペクティブ解析のため該当せず.

<インフォームド・コンセントの手続き>　なし

<代諾者の選定方針>　なし

１７.　研究対象者に緊急かつ明白な生命の危機が生じている状況における研究を実施しようとする場合

レトロスペクティブ解析のため該当せず.

１８.　研究対象者等の経済的負担および謝礼

レトロスペクティブ解析のため該当せず.

<経済的負担>　なし

<謝礼>　なし

１９.　重篤な有害事象が発生した際の対応　（侵襲を伴う研究の場合）

レトロスペクティブ解析のため該当せず.

２０.　健康被害に対する補償　（侵襲を伴う研究の場合）

レトロスペクティブ解析のため該当せず.

２１.　研究対象者への研究実施後における医療の提供

レトロスペクティブ解析のため該当せず.

２２.　研究結果（偶発的所見を含む）の取扱い

レトロスペクティブ解析のため該当せず.

２３.　当該業務内容及び委託先の監督方法（業務委託をする場合のみ）

該当せず.

２４.　研究対象者から取得された試料・情報について、研究対象者等から同意を受ける時点では特定されない将来の研究のために用いられる可能性又は他の研究機関に提供する可能性がある場合には、その旨と同意を受ける時点において想定される内容

該当せず.

２５.　侵襲（軽微な侵襲を除く）を伴う研究であって介入を行うものを実施する場合にはモニタリング及び監査の実施体制及び実施手順

該当せず.

別添１

個人情報の取扱い（第三病院）

<table>
<tr><td rowspan="2">Ⅰ匿名化の状況</td><td colspan="4">匿名化（対応表の作成）の有無</td></tr>
<tr><td colspan="4">■匿名化を行う
⇒対応表の作成有無
■　作成する
□　作成しない
□　その他（　　　　　　　　　　　　　　　　　）
□匿名化を行わない（理由：　　　　　　　　　　　　　　　　　）</td></tr>
<tr><td rowspan="7">Ⅱ対応表の管理
㊟対応表の管理・廃棄責任者は個人情報管理者</td><td colspan="4">①　対応表の管理</td></tr>
<tr><td>個人情報分担管理者</td><td>所属：救急医学講座
氏名：光永敏哉</td><td>個人情報管理補助者（匿名化作業）</td><td>所属：救急医学講座
氏名：大谷圭</td></tr>
<tr><td colspan="4">②　対応表の保管場所（　救急部カンファレンスルーム内　）</td></tr>
<tr><td colspan="4">保管方法
□外部と切り離された PC を使用して、外部記憶媒体に保存し、鍵の掛かる保管庫に厳重に保管する
■紙媒体に記録し、鍵の掛かる保管庫に厳重に保管する
□その他（　　　　　　）</td></tr>
<tr><td colspan="4">③　対応表の廃棄方法</td></tr>
<tr><td colspan="4">□外部記憶装置媒体内データを消去した上で、媒体を物理的に破壊
■個人情報に注意しシュレッダーで裁断
□その他（　　　　　）</td></tr>
<tr><td colspan="4"></td></tr>
<tr><td rowspan="4">Ⅲ情報の管理
㊟情報の管理責任は研究代表者</td><td colspan="4">①　保管場所　（　救急医学講座内　）</td></tr>
<tr><td colspan="4">保管方法
□外部と切り離された PC を使用して、外部記憶媒体に保存し、鍵の掛かる保管庫に厳重に保管
■外部と切り離された PC で、ログイン時及びファイル開封時にﾊﾟｽﾜｰﾄﾞによりｱｸｾｽ制限の掛かった PC 内のファイルに記録
□カルテ内
□その他（　　　　　　　　　　　　　　　　　　　　）</td></tr>
<tr><td colspan="4">②　廃棄方法</td></tr>
<tr><td colspan="4">□外部記憶装置媒体内データを消去した上で、物理的に破壊
■PC 内のデータ消去ソフトを使用し消去
□その他（　　　　　　　　　）</td></tr>
</table>

Ⅳ安全管理措置	■ﾊﾟｽﾜｰﾄﾞ設定によりｱｸｾｽ権保有者以外は PC へのｱｸｾｽ不能（ｱｸｾｽ制御）<br>（ｱｸｾｽ権保有者：光永敏哉）<br>■不正ソフトウェア防止ソフト導入済みの PC を使用<br>■外部との接続の無い PC を使用<br>□入退室管理の実施<br>■盗難・紛失の防止（□鍵の掛かるキャビネットへの保管　■持ち歩きの厳禁）<br>□その他（　　　　　　　　　　　　　　　　　　　　　　　）
Ⅴ 情報（対応表含む）の保管期間	■少なくとも、当該研究の終了について報告された日から５年を経過した日又は当該研究の結果の最終の公表について報告された日から３年を経過した日のいずれか遅い日までの期間<br>□その他（　　　　　　　　　　　　　　　　　　　　　　　　　　　）

別添１

個 人 情 報 の 取 扱 い（柏病院）

<table>
<tr><td rowspan="2">Ⅰ匿名化の状況</td><td colspan="4">匿名化（対応表の作成）の有無</td></tr>
<tr><td colspan="4">■匿名化を行う
⇒対応表の作成有無
■ 作成する
☐ 作成しない
☐ その他（ ）
☐匿名化を行わない（理由： ）</td></tr>
<tr><td rowspan="7">Ⅱ対応表の管理
<u>㊟対応表の管理・廃棄責任者は個人情報管理者</u></td><td colspan="4">② 対応表の管理</td></tr>
<tr><td>個人情報分担管理者</td><td>所属：救急医学講座
氏名：光永敏哉</td><td>個人情報管理補助者（匿名化作業）</td><td>所属：救急医学講座
氏名：長谷川意純</td></tr>
<tr><td colspan="4">④ 対応表の保管場所（ 救急部スタッフルーム内 ）</td></tr>
<tr><td colspan="4">保管方法
☐外部と切り離された PC を使用して、外部記憶媒体に保存し、鍵の掛かる保管庫に厳重に保管する
■紙媒体に記録し、鍵の掛かる保管庫に厳重に保管する
☐その他（ ）</td></tr>
<tr><td colspan="4">⑤ 対応表の廃棄方法</td></tr>
<tr><td colspan="4">☐外部記憶装置媒体内データを消去した上で、媒体を物理的に破壊
■個人情報に注意しシュレッダーで裁断
☐その他（ ）</td></tr>
<tr><td colspan="4"></td></tr>
<tr><td rowspan="4">Ⅲ情報の管理
<u>㊟情報の管理責任は研究代表者</u></td><td colspan="4">③ 保管場所 （ 救急医学講座医局内 ）</td></tr>
<tr><td colspan="4">保管方法
☐外部と切り離された PC を使用して、外部記憶媒体に保存し、鍵の掛かる保管庫に厳重に保管
■外部と切り離された PC で、ログイン時及びファイル開封時にﾊﾟｽﾜｰﾄﾞによりｱｸｾｽ制限の掛かった PC 内のファイルに記録
☐カルテ内
☐その他（ ）</td></tr>
<tr><td colspan="4">④ 廃棄方法</td></tr>
<tr><td colspan="4">☐外部記憶装置媒体内データを消去した上で、物理的に破壊
■PC 内のデータ消去ソフトを使用し消去
☐その他（ ）</td></tr>
</table>

Ⅳ安全管理措置	■ﾊﾟｽﾜｰﾄﾞ設定によりｱｸｾｽ権保有者以外は PC へのｱｸｾｽ不能（ｱｸｾｽ制御）（ｱｸｾｽ権保有者：光永敏哉）<br>■不正ソフトウェア防止ソフト導入済みの PC を使用<br>■外部との接続の無い PC を使用<br>□入退室管理の実施<br>■盗難・紛失の防止（□鍵の掛かるキャビネットへの保管　■持ち歩きの厳禁）<br>□その他（　　　　　　　　　　　　　　　　　　　　　）
Ⅴ 情報（対応表含む）の保管期間	■少なくとも、当該研究の終了について報告された日から５年を経過した日又は当該研究の結果の最終の公表について報告された日から３年を経過した日のいずれか遅い日までの期間<br>□その他（　　　　　　　　　　　　　　　　　　　　　　　　　　）

別添 2

試料・情報の授受に関する記録の作成及び保管について

本研究において、共同研究機関／試料・情報の提供のみを行う者との間で取り扱う試料・情報の授受の記録作成及び保管については、以下のように定める。尚、各共同研究機関及び各機関の研究責任者／試料・情報の提供のみを行う者とその所属機関の一覧については、研究計画書のとおり。

提供元となる機関での取扱い		提供を受ける機関での取扱い	
①提供する情報	●スコアリングに必要な情報：①意識状態、②脈拍数、③呼吸数、④収縮期血圧 or MAP、⑤体温、⑥SpO2、⑦酸素投与、⑧年齢<br>●その他患者情報：①来院月、②来院時刻、③年齢、④性別、⑤診断カテゴリー（総合内科・呼吸器内科・循環器内科・消化器肝臓内科・神経内科・血液腫瘍内科・糖尿病代謝内分泌内科・腎臓高血圧内科・膠原病内科・外科・整形外科・脳神経外科・形成外科・泌尿器科・耳鼻咽喉科・その他）、⑥転帰**（帰宅・一般床入院・ICU 入院・死亡）、⑦採血項目（CRP・アルブミン値・血液ガス）**	⑫提供を受ける情報（項目）	①　に同じ
②情報の取得経緯	診療過程で取得されるものである。	⑬提供元機関での情報の取得経緯	②に同じ
③提供する情報の由来者	対応表を作成し、匿名化を施したものを提供する。但し、対応表の提供は伴わない。	⑭提供を受ける情報の由来者	提供を受けた情報そのものを保管。
④提供記録の作成時期	継続審査または終了時に一括で作成	⑮収受記録の作成時期	継続審査または終了時に一括で作成
⑤提供記録の記録媒体	救急医学講座に設置された管理用 PC 内の電子ファイル	⑯収受記録の記録媒体	千葉大学予防医学センターオミクス応用解析研究室内に設置された管理用 PC 内の電子ファイル
⑥提供記録の作成者	東京慈恵会医科大学　救急医学講座　光永敏哉（助教）	⑰収受記録の作成者	千葉大学　予防医学センター　関根章博（教授）

**⑦提供記録の代用書類**	無	**⑱収受記録の代用書類有無**	無
**⑧提供記録の保管場所**	東京慈恵会医科大学　救急医学講座内の記録管理用キャビネット	**⑲収受記録の保管場所**	千葉大学予防医学センターオミクス応用解析研究室内記録管理用キャビネット
**⑨対応表の管理責任者･管理場所**	研究個人情報分担管理者の下管理する。<br>紙媒体に記録し、鍵のかかる保管庫に厳重に保管する。	**⑳提供を受ける機関･責任者**	千葉大学　予防医学センター　関根章博（教授）
**⑩提供先機関･責任者**	東京慈恵会医科大学　救急医学講座　光永敏哉（助教）	**㉑提供元機関･責任者**	千葉大学　予防医学センター　関根章博（教授）
**⑪提供記録の保管期間**	少なくとも提供予定日（2018 年 8 月）から 3 年。	**㉒収受記録の保管期間**	少なくとも本研究の終了報告（2020年 3 月末予定）から 3 年。
