# Supplementary material for: Comparison of the National Early Warning Score (NEWS) and the Modified Early Warning Score (MEWS) for predicting admission and in-hospital mortality in elderly patients in the pre-hospital setting and in the emergency department
Source: PeerJ. 2019 May 16;7:e6947. doi: 10.7717/peerj.6947 (PMC6526008; doi:10.7717/peerj.6947)
Supplement: Supplemental Information 3 [file peerj-07-6947-s003.docx]

人を対象とする医学系研究に関する倫理指針（改訂版H29.7.3）Ethical guidelines on medical research for people (revised version H.29.7.3 )

**研究実施計画書Research implementation plan**

**第１版 2018年5月10日First** **edition 10 May, 2018**

**第７版201９年１月１８日Seventh** **Edition** **18 January,** **2019**

**課題名「救急搬送された高齢患者の予後予測スコアは、死亡率・入院率や救急部滞在時間と関連性があるか」Subject "The relevance between prognostic score** **of elderly patients** **who have been ambulance, and the** **mortality, hospitalization** **rates** **and emergency department stay time."**

**１．1.**       **研究の組織・実施体制**（研究機関の名称及び研究者等の氏名・役割を含む。）**Organization / implementation system of** research (including names of research institutes and names and roles of researchers etc.)

**〈研究代表者〉<Research leader>**

光永敏哉 東京慈恵会医科大学 救急医学講座 助教 研究計画の立案・データ管理者責任者Toshiya Mitsunaga Tokyo Jikei University School of Medicine Emergency Medicine course Assistant Professor Planning and planning data managers

**〈研究分担者〉<Study sharing person>**

武田 聡 東京慈恵会医科大学 救急医学講座 教授 研究計画アドバイザーSatoru Takeda Tokyo Jikei University School of Medicine Emergency Medicine Course Professor Research Planning Advisor

卯津羅 雅彦 東京慈恵会医科大学 救急医学講座 教授 研究計画アドバイザーMasahiko Uunazura Department of Emergency Medicine, Department of Medicine, Jikei University School of Medicine Research Plan Advisor

大谷 圭 東京慈恵会医科大学 救急医学講座 准教授Kei Otani Associate Professor, Department of Emergency Medicine, Jikei University School of Medicine 　　 研究計画アドバイザー・データ収集責任者Research plan advisor · Data collection supervisor

長谷川 意純 東京慈恵会医科大学 救急医学講座 助教 データ収集担当者Atsuto Hasegawa Tokyo Jikei University School of Medicine Emergency Medicine course Assistant Professor Data Collector

**〈共同研究者〉<Collaborator>**

関根 章博 千葉大学 予防医学センター 教授 データ解析担当者・研究計画アドバイザーChief Sekine Chiba University Center for Preventive Medicine Professor Data Analyst Personnel · Research Plan Advisor

**２．2.**       **研究の目的及び意義Purpose and significance of research**

**<目的><Purpose>** 　 近年増加傾向にある高齢救急患者のRapid Acute Physiology Score (RAPS)/ Rapid Emergency Medicine Score (REMS)/National Early Warning Score(NEWS)/Rapid Acute Physiology Score of elderly emergency patients in recent years an increasing trend (RAPS) / Rapid Emergency Medicine Score (REMS) / National Early Warning Score (NEWS) / Modified Early Warning Score(MEWS)と**死亡率**・入院**率**や救急部滞在時間との関連性を明らかにする。We clarify the relationship between Modified Early Warning Score (MEWS) and death rate · hospitalization rate and staying time in emergency department .

**<意義><Significance>**

近年，多くの医療機関における救急外来の過剰な混雑状態が問題となっている。In recent years, excessive congestion state of emergency department in a number of medical institutions has become a problem. 中でも，救急現場での軽症患者数の増加により，重症救急患者への対応の遅れや患者の訴えの見逃しだけでなく，救急医療従事者の過重労働など問題が山積している。Among them, due to the increase in the number of patients with mild illness in emergency situations, problems such as delays in dealing with serious emergency patients and overlooking patient complaints, as well as emergency medical workers' overwork labor, etc. are piling up. 救急隊による年間の救急搬送件数は1990年に約270万人であったのが，2000年に約400万人，2012年には約520万人に増加しており，これは国民の24人に1人が救急隊によって搬送されたことになる。The number of annual emergency transportation by emergency services was increased, about 2.7 million in 1990, 4 million in 2000 and 5.2 million in 2012. 搬送患者の中でも，特に軽症者の増加が著しい。Among patients being transported, the number of people with a particularly mild disease has increased remarkably. また，平成24年度119番通報入電から病院等に収容するまでの平均時間は全国で38.7分であるが，東京都では平成24年度54.9分と47都道府県中最下位となっており，救急外来受診の適正化と速やかな救急要請の受け入れは急務である。In addition, the average time from the 119th report reception call to the hospital etc. in the hospital etc. is 38.7 minutes nationwide, but the Tokyo metropolitan area is the lowest among 47 prefectures, 54.9 minutes in 2012, the emergency outpatient acceptance of optimization and rapid emergency request of the visit is Ru urgent need.

搬送される患者のうち、65歳以上の高齢者の数がこの10年間で激増している。Among the patients being transported, the number of elderly people aged 65 or over has increased dramatically in the past 10 years. 高齢患者では、主訴の曖昧さから時に診察が困難であり、検査数の増加や複数の診療科にコンサルトするために救急部滞在時間が長くなってしまう。In elderly patients, diagnosis is difficult at times due to ambiguity of the chief complaint, and the number of examinations increases and the duration of staying in the emergency department becomes longer to consult to multiple departments. 救急外来における高齢患者のトリアージをいかに素早く行うかが、救急部の円滑な運営にとっても必要不可欠である。How quickly triage of elderly patients in emergency outpatient is necessary is also essential for the smooth operation of the emergency department.

Rapid Acute Physiology Score (RAPS)/ Rapid Emergency Medicine Score (REMS)/National Early Warning Score(NEWS)/Rapid Acute Physiology Score (RAPS) / Rapid Emergency Medicine Score (REMS) / National Early Warning Score (NEWS) / Modified Early Warning Score(MEWS)はベッドサイドで患者の急変予測を行うツールとして、世界各地で導入された。The Modified Early Warning Score (MEWS) were introduced worldwide as a tool to predict sudden change of the patient on the bedside. これらスコアの導入によって院内の死亡率が低下したり、緊急のICU入室率を低下させることができた。Mortality of hospital by the introduction of these scores was able to reduce or decrease the emergency of ICU admission rate.

我々は、これらのスコアを用いて、救急外来に搬送された高齢患者の入院や救急部滞在時間との関連性を解析し、高齢救急患者のトリアージツールとしての有用性を検討する。We used the these scores, to analyze the association between hospitalization and emergency department stay time of elderly patients who have been transported to the emergency room, to examine the usefulness as a triage tool of elderly emergency patients.

**３．3.**       **研究の方法Method of research**

**・研究デザイン：**Retrospective Observational Study**· Research design:** Retrospective Observational Study

**・研究対象者：**2017年４月1日から2018年３月31日までの期間に東京慈恵会医科大学附属第三病院救急部と東京慈恵会医科大学附属柏病院救命救急センターを受診した患者のうち以下の症例を対象とする。**And research subjects:** 201 was admitted to Jikei University School of Medicine University third hospital emergency department and the Jikei University School of Medicine University Kashiwa Hospital emergency center during the period from 7 April 1 to March 31, 2018 patients The following cases are targeted.

**【Inclusion criteria:】【Inclusion criteria:】**

1)1)        救急搬送された65歳以上のすべての患者All patients over 65 who were emergency transported

**・研究方法：·research method:**

　 ●本研究は千葉大学との共同研究であり、本学は観察および検査等で得られた情報を千葉大学と共有し解析を行う。● This research is a collaborative research with Chiba University, and the university shares information obtained by observation and inspection etc. with Chiba University and analyzes it.

●Rapid Acute Physiology Score (RAPS)● Rapid Acute Physiology Score (RAPS)

|  | 00 | +1+1 | +2+2 | +3+3 | +4+4 |
| --- | --- | --- | --- | --- | --- |
| 脈拍数Pulse rate | 70-10970-109 |  | 55-6955-69  110-139110-139 | 40-5440-54  140-179140-179 | 39以下39 or less  180以上Over 180 |
| MAPMAP | 70-10970-109 |  | 50-6950-69  110-129110 - 129 | 130-159130-159 | 49以下Below 49  160以上Over 160 |
| 呼吸数Respiratory rate | 12-2412-24 | 10-1110-11  25-3425-34 | 6-96-9 | 35-4935-49 | 5以下5 or less  50以上50 or more |
| GCSGCS | 14以上14 or more | 11-1311-13 | 8-108-10 | 5-75-7 | 4以下4 or less |

●Rapid Emergency Medicine Score (REMS)● Rapid Emergency Medicine Score (REMS)

|  | 00 | +1+1 | +2+2 | +3+3 | +4+4 | +5+5 | +6+6 |
| --- | --- | --- | --- | --- | --- | --- | --- |
| 年齢age | 45以下45 or less |  | 45-5445-54 | 55-6455-64 |  | 65-7465-74 | 74以上74 or more |
| 脈拍数Pulse rate | 70-10970-109 |  | 55-6955-69  110-139110-139 | 40-5440-54  140-179140-179 | 39以下39 or less  179以上Over 179 |  |  |
| MAPMAP | 70-10970-109 |  | 50-6950-69  110-129110 - 129 | 130-159130-159 | 49以下Below 49  159以上159 or more |  |  |
| 呼吸数Respiratory rate | 12-2412-24 | 10-1110-11  25-3425-34 | 6-96-9 | 35-4935-49 | 5以下5 or less  49以上49 or more |  |  |
| GCSGCS | 14 or 1514 or 15 | 11-1311-13 | 8-108-10 | 5-75-7 | 3 or 43 or 4 |  |  |
| SpO2SpO 2 | 89以上More than 89 | 86-8986-89 |  | 75-8575-85 | 75以下75 or less |  |  |

●National Early Warning Score(NEWS)● National Early Warning Score (NEWS)

|  | 33 | 22 | 11 | 00 | 11 | 22 | 33 |
| --- | --- | --- | --- | --- | --- | --- | --- |
| 呼吸数Respiratory rate | 8以下8 or less |  | 9-119-11 | 12-2012-20 |  | 21-2421-24 | 25以上25 or more |
| SpO2SpO 2 | 91以下91 or less | 92-9392-93 | 94-9594-95 | 96以上Over 96 |  |  |  |
| 酸素投与Oxygen administration |  | YesYes |  | NoNo |  |  |  |
| 体温Body temperature | 35以下35 or less |  | 35.1-36.035.1-36.0 | 36.1-38.036.1-38.0 | 38.1-39.038.1-39.0 | 39.1以上39.1 and above |  |
| 収縮期血圧Systolic blood pressure | 90以下90 or less | 91-10091-100 | 101-110101-110 | 111-219111-219 |  |  | 220以上Over 220 |
| 脈拍数Pulse rate | 40以下40 or less |  | 41-5041-50 | 51-9051 - 90 | 91-11091-110 | 111-130111-130 | 131以上More than 131 |
| 意識覚醒Consciousness arousal |  |  |  | AA |  |  | V,P, or UV, P, or U |

　 ●Modified Early Warning Score(MEWS)● Modified Early Warning Score (MEWS)

| PointsPoints | 33 | 22 | 11 | 00 | 11 | 22 | 33 |
| --- | --- | --- | --- | --- | --- | --- | --- |
| 体温Body temperature  °C° C |  | 35以下35 or less |  | 35.1-38.435.1-38.4 |  | 38.5以上38.5 and above |  |
| 脈拍数Pulse rate  bpmbpm |  | 39以下39 or less | 40-5040-50 | 51-10051-100 | 101-110101-110 | 111-129111-129 | 130以上Over 130 |
| 収縮期血圧Systolic blood pressure  mmHgmmHg | 70以下70 or less | 71-8071-80 | 81-10081-100 | 101-199101-199 |  | 200以上Over 200 |  |
| 呼吸数Respiratory rate |  | 8以下8 or less | 99 | 10-1810-18 | 19-2019-20 | 21-2921-29 | 30以上30 or more |
| AVPUAVPU |  |  |  | AA | VV | PP | UU |

●電子カルテから、**救急外来ならびに救急隊が病院前で記載した**上記スコアに必要な①意識状態、②脈拍数、③呼吸数、④収縮期血圧or MAP、⑤体温、⑥SpO2、⑦酸素投与、⑧年齢に関する情報を抽出する。● From the electronic medical record , the emergency outpatient as well as the emergency room described in front of the hospital ① awareness state ② conscious state, ② pulse rate, ③ breathing rate, ④ systolic blood pressure or MAP , ⑤ body temperature, ⑥ SpO2 , ⑦ oxygen administration , ⑧ Extract information on age .

●患者情報として、①来院月、②来院時刻、③年齢、④性別、⑤診断カテゴリー（総合内科・呼吸器内科・循環器内科・消化器肝臓内科・神経内科・血液腫瘍内科・糖尿病代謝内分泌内科・腎臓高血圧内科・膠原病内科・外科・整形外科・脳神経外科・形成外科・泌尿器科・耳鼻咽喉科・その他）、⑥既往歴、⑦認知症の有無・程度、⑧ADL、⑨生活場所（他医療機関・福祉施設・自宅）、⑩転帰（帰宅・ICU入院・一般床入院・**28日死亡率**）、**⑩採血項目（CRP・アルブミン値・血液ガス）**を収集する。● Patient information includes: ① Visit Month, ② Visit Time, ③ Age, ④ Gender, ⑤ Diagnosis Category (Comprehensive Internal Medicine · Internal Medicine of Cardiovascular Medicine · Internal Medicine of Gastroenterology · Internal Medicine of Gastroenterology · Internal Medicine of Neurological Diseases · Internal Medicine of Hematology · Diabetes Metabolism Endocrine Internal Medicine · Kidney Hypertension Internal Medicine · Collagen Disease Internal Medicine · Surgery · Orthopedic Surgery · Neurosurgery · Plastic Surgery · Urology · Otolaryngology · Others) , ⑥ History, ⑦ Absence / degree of dementia, ⑧ ADL , ⑨ Living place medical institutions and welfare facilities and home), ⑩ outcome (return home · ICU hospitalization, general floor hospital, **28-day mortality rate),** to collect the **⑩ blood collection item (CRP · albumin level, blood gas).**

**・主要評価項目（Primary endpoint）：·** **Primary endpoint** **:**

●上記各スコアの点数と入院・滞在時間に相関関係があるか。● whether there is a correlation score and hospital-stay time of each of the above score.

**・実施場所：· Place of implementation:**

①①        東京慈恵会医科大学附属第三病院救急部Tokyojikeikai Medical University third hospital emergency department

②②        東京慈恵会医科大学附属柏病院救命救急センターTokyo Jikei Hospital Medical University Attached Kashiwa Hospital Critical Care and Emergency Center

**・統計学的解析：**本試験の評価項目として設定した種々の観察項目のデータのカテゴリーに従い，適切な統計解析を、統計ソフトを用いて比較検討する．**· Statistical analysis:** Compare and analyze appropriate statistical analysis using statistical software according to the categories of data of various observation items set as the evaluation items of this test. 統計解析は救急医学講座医局内（本院）または、千葉大学予防医学センターオミクス応用解析研究室内にて行う。Statistical analysis Emergency Medicine Lecture within the medical office (the Museum) or, carried out at Chiba University Preventive Medicine Center omics applied analysis laboratory. 著名化された情報は、パスワード付きUSBメモリーに保管し、研究代表者が千葉大学へ持参する。The famous information is kept in a USB memory with password, and the research representative brings it to Chiba University.

**４．4.**       **目標症例数及び予定期間Target case number and planned period**

**<目標症例数><Target number of cases>**

参加施設：2施設Participating facilities: 2 facilities

目標対象者：2500例Target Subject: 2500 cases

**<症例数の設定根拠><Rationale for setting the number of cases>**

昨年度東京慈恵会医科大学附属第三病院救急部の救急車収容台数は約3,500台で東京慈恵会医科大学附属柏病院救命救急センターの救急車収容台数は約5,000台であった。Ambulance accommodation number of last year Jikei University School of Medicine University third hospital emergency department is about 3, at 500 Jikei University School of Medicine ambulance accommodation number of University Kashiwa Hospital emergency center was about 5, 000 units. H29年度総務省消防庁のまとめでは、全救急搬送患者のうち57.2%が65歳以上であった。In H29_nendo Ministry of Internal Affairs and Communications summary of the Fire and Disaster Management Agency, 57.2% of all ambulance patients were at least 65 years of age. 従って、予想される65歳以上の患者数は8,500(台)×1(年)×0.5＝4,250例。Therefore, the expected number of patients over 65 years old was 8 , 500 ( units ) × 1 ( year ) × 0.5 = 4,250 cases . データ欠損例などが除外されると予想すると, 4,250×0.6=2,550であり,目標症例数は2,500例と設定した。If it is anticipated that examples of data loss will be excluded, it is 4,250 x 0.6 = 2,550 , and the target number of cases is set to 2,500 cases.

**<研究期間><Research Period>**

倫理委員会承認日より2020年3月31日迄From the Ethics Committee approval date until March 31, 2020

調査対象期間：2017年4月1日〜2018年3月31日Survey Period: 201 7 April 1, 2018 March 31, 2008

**５．5.**       **研究対象者の選定方針Selection policy of study subject**

**<登録基準><Registration criteria>** 　 2017年4月1日〜2018年3月31日に東京慈恵会医科大学附属第三病院救急部ならびに東京慈恵会医科大学附属柏病院救命救急センターに救急車で搬送された65歳以上のすべての患者。All patients 2017 April 65 years of age or older, which has been transported by ambulance to the Jikei University School of Medicine University third hospital emergency department as well as the Jikei University School of Medicine University Kashiwa Hospital emergency center 1, 2006 to March 31, 2018 .

**<除外基準><Exclusion criteria>** 　 なしNone

**６．6.**       **研究の科学的合理性の根拠Rationale for scientific rationality of research**

①①        Rapid Acute Physiology Score (RAPS)/ Rapid Emergency Medicine Score (REMS)/ National Early Warning Score (NEWS)/ Modified Early Warning Score (MEWS)はベッドサイドで患者の急変予測を行うツールとして、世界各地で導入され、これまでにアメリカにおいてModified Early Warning Scoreと入院との関連性について比較した研究が報告されているが、高齢化が進む本邦における報告はない。Rapid Acute Physiology Score (RAPS) / Rapid Emergency Medicine Score (REMS) / National Early Warning Score (NEWS) / Modified Early Warning Score (MEWS) is a tool for predicting sudden change in patients on the bedside, Although studies comparing the relationship between Modified Early Warning Score and hospitalization in the United States have been reported so far, there is no report in Japan as the aging progresses. 本研究では、複数のScoreについて高齢患者の入院や救急部滞在時間との関連性について解析する。In this study, we analyze the relationship between multiple scores of hospitalization of elderly patients and the duration of stay in the emergency department. 上記スコアが入院予測スコアとして有用であれば、今後救急外来における高齢患者の方針決定までの時間の短縮や救急外来混雑の緩和につながる可能性がある。If the above score is useful as a hospital prediction score, there is a possibility that it will shorten the time to policy decision of elderly patients in the emergency outpatient in the future and alleviate emergency outpatient congestion.

**７．7.**       **インフォームド・コンセントを受ける手続等Procedures to receive informed consent**

**<インフォームド・コンセントの方法><Informed consent method>**

本研究は，人を対象とする医学系研究に関する倫理指針の規定により，研究対象者からインフォームド・コンセントを受けることを必ずしも要しないと判断されるが，当該臨床研究の目的を含む研究の実施についての情報公開を行う．This research is judged not necessarily to receive an informed consent from the research subject by the provision of ethical guidelines concerning research on medicine research targeting human beings, but it is considered that research of the research including the purpose of the clinical research Disclose information on implementation.

本研究では倫理委員会で承認の得られた文書を救急外来にポスター掲示すると同時にホームページ上に公開することで情報公開を行うこととし，研究が実施または継続されることについて研究対象者が拒否できる機会を保障する．In this research, we will post a document that was approved by the Ethics Committee in the emergency outpatient and publish it on the website at the same time to disclose the information, and the research subject refused that the research will be conducted or continued Ensure opportunities to do.

なお，情報公開文書には以下の事項を含めることとする．Incidentally, the information disclosure document shall include the following items.

1.研究概要（名称・目的・研究期間など）1. Outline of research (Name, purpose, research period, etc.)

2.研究に利用する情報の項目2. Items of information used for research

3.他の機関へ情報等を提供する方法3. How to provide information etc. to other agencies

4.研究計画書および研究の方法に関する資料を入手または閲覧できる旨、並びにその入手・閲覧方法4. A statement that you can obtain or view materials on the research plan and research method, and how to obtain / view it

5.情報利用を拒否できる旨5. Ability to refuse information use

6.研究代表者の氏名6. Name of the research representative

**<試料・情報の授受に関する記録の作成及び保管について><** **About creation and storage of records relating to sample / information transfer** **>**

本研究は，共同研究機関と情報の授受があるため，情報の提供元と提供先で別添2「資料・情報の授受に関する記録の作成および保管について」のとおりとする．Because this research has information exchange with the collaborative research institution, please refer to Appendix 2 "About creating and keeping records on materials and information transfer" at the source and destination of information.

**８．8.**       **個人情報等の取扱い（匿名化する場合にはその方法を含む）Handling of personal information, etc.** **(including its method when making it anonymous)**

**<個人情報等の取扱い><Handling of personal information, etc.>**

本研究に係る研究対象者の個人情報は，「学校法人慈恵大学 個人情報保護に関する規定」，「個人情報の取得・利用ならびに第三者提供に関する細則」，「医学系研究（人を対象とする医学系研究に関する倫理指針およびヒトゲノム・遺伝子解析研究に関する倫理指針に基づく研究）における個人情報保護のための安全対策管理措置手順書」および「人を対象とする医学系研究に関する倫理指針」を遵守して取り扱う．Personal information of the subjects of research pertaining to this research is "Personal Information Protection of School Corporation Jiayu University", "Detailed Regulations Concerning Acquisition and Use of Personal Information and Provision of Third Parties", "Medical Research (Targeting People abiding the ethical guidelines "for medicine to target safety measures management measures order form" and "people for the protection of personal information in the study) based on the ethical guidelines for ethical guidelines and the human genome, genetic analysis research on medicine Protect and handle it.

解析結果は，研究対象者にプライバシー上の不利益が生じないよう，適切に匿名化されていることを確認した上で公表する．The results of analysis should be announced after confirming that they are appropriately anonymized so that research subjects will not suffer from privacy disadvantages. 研究代表者は、試料・情報の取得後、速やかに個人情報分担管理者に渡し匿名化を依頼する。After acquiring the sample / information, the research representative promptly requests the personal information sharing manager for anonymization. 取り扱う匿名化方法・対応表の管理方法、安全管理体制の詳細は、別添１の通りとする。Details of the anonymization method and handling correspondence table management method and safety management system are as shown in Attachment 1.

本研究に係る研究対象者の特定の個人を識別できる情報は，他の研究機関と共有しない．Information that can identify a specific individual of the research subject pertaining to this research is not shared with other research institutes.

**<個人情報保護相談窓口><Personal information protection consultation window>**

附属第三病院：03-3480-1151内線：3710Attached Third Hospital: 03-3480-1151 Extension: 3710

附属柏病院：04-7164-1111内線：2183Attached Kashiwa Hospital: 04-7164-1111 Ext. Line: 2183

対応時間：午前9時〜午後５時/休診日を除くCorrespondence time: 9 am - 5 pm / except for closed days

**９．9.**       **予測されるリスク及び利益Predicted risks and benefits**

**<有害事象・副作用><Adverse events / side effects>**

なしNone

**<リスクを最小化される対策><Measures to minimize risks>**

　　 なしNone

**<利益><Profit>**

本研究への参加することによる研究対象者に直接の利益は生じない．There is no direct benefit to the research subject by participating in this research. 研究成果により将来の救急医療の進歩に貢献できる可能性がある．There is a possibility that contribution to future advancement of emergency medical care can be contributed by research results.

**１０．10.** **試料・情報の保管及び廃棄の方法Method of storage and disposal of samples and information**

本研究に係る研究対象者の情報は，人体から取得された情報等の保管に関する手順書を尊守して取り扱う．The information on the subjects of research pertaining to this research shall be handled in accordance with the procedure manual concerning the storage of information and the like acquired from the human body.

1)1)        保管する情報：申請書類の控え，学長からの通知文書，情報の提供に関する記録，対応表，覚書Information to be kept: a copy of the application document, a notice from the president, a record on provision of information, correspondence chart , memorandum of understanding

2)2)        保管方法：別添1 個人情報の取り扱いに記載の通り．Storage method: As described in Attachment 1 Handling of personal information.

3)3)        破棄方法：別添1 個人情報の取り扱いに記載の通り．Disposal method: As described in Attachment 1 Handling of personal information.

4)Four)        保管期間：別添1 個人情報の取り扱いに記載の通り．Storage period: As described in Attachment 1 Handling of personal information.

5)Five)        本研究は、他機関と試料・情報の授受があるため、試料・情報の提供元と提供先との間で別添２の「試料・情報の授受にかんする記録の作成及び保管について」の通りとする。In this research, as sample and information are exchanged with other institutions, as described in Attachment 2 "Preparation and storage of records concerning sample / information exchange" between suppliers and suppliers of sample / information .

**１１．11.** **研究機関の長への報告内容及び方法Report content and method to the head of the research organization**

1)1)        研究代表者は、研究を実施（研究計画書を変更して実施する場合を含む。以下同じ。）しようとするときは、あらかじめ研究計画書を作成し、学長及び第三病院病院長・柏病院病院長の許可を受ける。When planning to carry out research (including cases where the research plan is changed and executed, the same applies below), the research representative prepares a research plan in advance, and the president and the third hospital hospital director · Kashiwa hospital Get permission of the hospital manager .

2)2)        研究代表者は、研究の倫理的妥当性若しくは科学的合理性を損なう事実若しくは情報又は損なうおそれのある情報であって研究の継続に影響を与えると考えられるものを得た場合には、遅滞なく、学長及び第三病院病院長・柏病院病院長に対して報告し、必要に応じて、研究を停止し、若しくは中止し、又は研究計画書を変更する。If a research representative obtains information that may impair the ethical validity or scientific rationality of the research or information that may be damaged and believed to affect the continuation of the research, , The President and the Head of the 3rd Hospital Hospital and the Head of the Kashiwa Hospital Hospital . If necessary, suspend or stop the research, or change the research plan.

3)3)        研究代表者は、研究の実施の適正性若しくは研究結果の信頼を損なう事実若しくは情報又は損なうおそれのある情報を得た場合には、速やかに学長及び第三病院病院長・柏病院病院長に報告し、必要に応じて、研究を停止し、若しくは中止し、又は研究計画書を変更する。If the research leader got information on the suitability of the research or information that may impair the credibility of the research results or information that may be damaged or lost, it will promptly report to the president and the head of the third hospital hospital and Kashiwa hospital hospital If necessary, stop or stop the research, or change the research plan.

4)Four)        研究代表者は、年に1回、研究の進捗状況及び研究の実施に伴う有害事象の発生状況、人体から取得された試料及び情報等の保管・管理状況について学長に報告する。The research representative reports to the President about the state of progress of research, the occurrence of adverse events accompanying the execution of research, and the status of storage and management of samples and information acquired from the human body once a year.

5)Five)        研究代表者は、研究を終了（中止の場合を含む。以下同じ。）したときは、学長及び第三病院病院長・柏病院病院長に必要な事項について報告する。When including the termination of research (including the case of cancellation, the same shall apply hereinafter), the research representative shall report to the president and the head of the third hospital hospital and Kashiwa hospital the necessary items. また、侵襲（軽微な侵襲を除く）を伴う研究であって介入を行うものについて、結果の最終の公表を行ったときは、遅滞なく研究機関の長へ報告する。Also, when conducting a study involving invasion (excluding minor invasion) and making the final public announcement of the results of intervention, report to the head of the research institution without delay.

**１２．12.** **研究の資金源と利益相反Funding sources of research** **and** **conflicts of interest**

**<研究の資金源><Funding source for research>**

この研究に関する経費は，研究代表者が所属する講座・研究室の研究費で賄われる．Expenses for this research will be covered by the research expenses of the lecture / laboratory to which the research representative belongs.

**<利益相反および個人の収益等><Conflicts of interest and individual profits>**

この研究は，特定の企業・団体との関与はない．This research has no involvement with specific companies / organizations. 研究の実施に当たり，研究の透明性および公平性の確保に努める．In conducting research, we strive to ensure transparency and fairness of research. なお，研究者は本学の利益相反管理規定を尊守し、利益相反管理委員会への手続きを行っている．Researchers respect the conflict of interest control provisions of the University and are conducting procedures to the Conflict of Interest Management Committee.

**１３．13.** **研究に関する情報公開の方法Information disclosure method on research**

介入を伴う研究ではないため，公開データベースへの登録は行わない．Since it is not a research involving intervention, it is not registered in the public database.

研究結果は、海外学会や英文誌へ発表する．The research results are presented to overseas academic societies and English journals.

**１４．14.** **研究対象者等及びその関係者からの相談等への対応Responding to consultation etc. from research subjects and related persons**

担当者：光永敏哉Person in charge: Toshiya Mitsunaga

電話番号：03-3433-1111（内線：3115）Phone number: 03-3433-1111 (extension: 3115)

**１５．15.** **代諾者等からインフォームド・コンセントを受ける手続Procedures** **to receive** **informed consent from substitutors**

レトロスペクティブ解析のため該当せず．Not applicable due to retrospective analysis.

**<インフォームド・コンセントの手続き>なし<Informed consent procedures>** **none**

**<代諾者の選定方針>なし<Selection policy of substitute person>** **none**

**１６．16.** **インフォームド・アセントの手続Procedure of informed ascent**

レトロスペクティブ解析のため該当せず．Not applicable due to retrospective analysis.

**<インフォームド・コンセントの手続き>なし<Informed consent procedures>** **none**

**<代諾者の選定方針>なし<Selection policy of substitute person>** **none**

**１７．17.** **研究対象者に緊急かつ明白な生命の危機が生じている状況における研究を実施しようとする場合When trying to conduct research in a situation where research subjects have an urgent and obvious life crisis occurring**

　　 レトロスペクティブ解析のため該当せず．Not applicable due to retrospective analysis.

**１８．18.** **研究対象者等の経済的負担および謝礼Economic burden** **and** **reward,** **such as research subjects**

レトロスペクティブ解析のため該当せず．Not applicable due to retrospective analysis.

**<経済的負担>なし<Economic burden>** **none**

**<謝礼>なし<Reward>** **none**

**１９．19.** **重篤な有害事象が発生した際の対応Correspondence when serious adverse event occurs** 　 **（侵襲を伴う研究の場合）(In the case of research involving invasion)**

レトロスペクティブ解析のため該当せず．Not applicable due to retrospective analysis.

**２０．20.** **健康被害に対する補償Compensation for health damage** 　 **（侵襲を伴う研究の場合）(In the case of research involving invasion)**

レトロスペクティブ解析のため該当せず．Not applicable due to retrospective analysis.

**２１．21.** **研究対象者への研究実施後における医療の提供Provide medical treatment after conducting research to the research subject**

レトロスペクティブ解析のため該当せず．Not applicable due to retrospective analysis.

**２２．22.** **研究結果（偶発的所見を含む）の取扱いHandling of research results (including accidental findings)**

レトロスペクティブ解析のため該当せず．Not applicable due to retrospective analysis.

**２３．23.** **当該業務内容及び委託先の監督方法（業務委託をする場合のみ）The business content and subcontractors of inspection methods** **(if** **the** **outsourcing** **only)**

該当せず．Not applicable.

**２４．24.** **研究対象者から取得された試料・情報について、研究対象者等から同意を受ける時点では特定されない将来の研究のために用いられる可能性又は他の研究機関に提供する可能性がある場合には、その旨と同意を受ける時点において想定される内容If there is a possibility of using the sample / information obtained from the research subject for future research not specified at the time of receiving agreement from the research subject etc. or possibly providing it to another research institution, What is assumed at the time of receiving agreement and consent**

　　 該当せず．Not applicable.

**２５．25.** **侵襲（軽微な侵襲を除く）を伴う研究であって介入を行うものを実施する場合にはモニタリング及び監査の実施体制及び実施手順In the case of conduct involving invasion (excluding minor invasion) and implementing interventions, the implementation and monitoring procedure and monitoring procedure**

該当せず．Not applicable.

**個Pieces** **人Man** **情Sentiment** **報A report** **のof** **取Take** **扱Treatment** **い（第三病院）Yes** **(third hospital)**

| **Ⅰ匿名化の状況Ⅰ Status of anonymization** | **匿名化（対応表の作成）の有無Presence or absence of anonymization (creation of correspondence table)** | | | |
| --- | --- | --- | --- | --- |
|  | ■匿名化を行う■ carry out the anonymous  ⇒対応表の作成有無⇒ Create correspondence table  ■作成する■ Create  □作成しない□ Do not create  □ その他（ ）□ Other ()  □匿名化を行わない（理由： ）□ Do not anonymize (Reason:) | | | |
| **Ⅱ対応表の管理Ⅱ Management of correspondence table**  ㊟対応表の管理・廃棄責任者は個人情報 Management and disposal of correspondence table Person in charge  管理者Administrator | **①①**   **対応表の管理Management of correspondence table** | | | |
|  | 個人情報分担Distribution of personal information  管理者Administrator | 所属：救急医学講座Affiliation: Emergency Medical Course  氏名：光永敏哉Name: Toshiya Mitsunaga | 個人情報管理Personal information management  補助者Assistant  （匿名化作業）(Anonymization work) | 所属：救急医学講座Affiliation: Emergency Medical Course  氏名：大谷圭Name: Kei Otani |
|  | **②②**  **対応表の保管場所（Storage** **location of** **correspondence table** **(**救急部カンファレンスルーム内**）**In the emergency room conference room **)** | | | |
|  | 保管方法Storage method  □外部と切り離されたPCを使用して、外部記憶媒体に保存し、□ Save it to an external storage medium using a PC separated from the outside,  鍵の掛かる保管庫に厳重に保管するKeep it strictly in the key storehouse.  ■紙媒体に記録し、鍵の掛かる保管庫に厳重に保管する■ Record it on paper medium and store it strictly in the key storehouse  □その他（ ）□ Other () | | | |
|  | **③③**  **対応表の廃棄方法Disposal method of correspondence table** | | | |
|  | □外部記憶装置媒体内データを消去した上で、媒体を物理的に破壊□ External storage device Physically destroy the medium after erasing the data in the medium  ■個人情報に注意しシュレッダーで裁断■ Cut by shredder with careful attention to personal information  □その他（ ）□ Other () | | | |
| **Ⅲ情報の管理Ⅲ** **Management of** **information**    ㊟情報の管理責任は研究代表者Management responsibility for information is researcher | **①①**   **保管場所Storage location** **（(** 　 救急医学講座内**）**Emergency medical course **)** | | | |
|  | 保管方法Storage method  □外部と切り離されたPCを使用して、外部記憶媒体に保存し、鍵の掛かる保管□ Save using a PC detached from the outside to an external storage medium, keep locked  庫に厳重に保管Strictly kept in storage  ■外部と切り離されたPCで、ログイン時及びファイル開封時にﾊﾟｽﾜｰﾄﾞによりｱｸｾｽ制限の掛かったPC内のファイルに記録■ A PC detached from the outside records it in a file in the PC where access is restricted by password when logging in and opening the file  □カルテ内□ In the medical record  □その他（）□ Other ( ) | | | |
|  | **②②**        **廃棄方法Disposal method** | | | |
|  | □外部記憶装置媒体内データを消去した上で、物理的に破壊□ Physically destroy data after deleting data in the external storage device medium  ■PC内のデータ消去ソフトを使用し消去■ Use data erasure software in PC to erase  □その他（□ Other ( 　　　　　 ）) | | | |
| **Ⅳ安全管理措置IV Safety management measures** | ■ﾊﾟｽﾜｰﾄﾞ設定によりｱｸｾｽ権保有者以外はPCへのｱｸｾｽ不能（ｱｸｾｽ制御）■ other than the access rights holders by the password setting is inaccessible to the PC (access control)  （ｱｸｾｽ権保有者：光永敏哉）( Access right holder: Toshiya Mitsunaga )  ■不正ソフトウェア防止ソフト導入済みのPCを使用■ use unauthorized software prevention software already introduced PC  ■外部との接続の無いPCを使用■ Use a PC without external connection  □入退室管理の実施□ Implement entry control  ■盗難・紛失の防止（□鍵の掛かるキャビネットへの保管 ■持ち歩きの厳禁）■ Prevention of theft and loss (□ storage in a cabinet where keys are hooked ■ prohibition of carrying around)  □その他（）□ Other ( )              ）) | | | |
| **ⅤⅤ** **情報（対応表含む）の保管期間Storage period of information (including correspondence table)** | ■少なくとも、当該研究の終了について報告された日から５年を経過した日又は当該研究の結果の最終の公表について報告された日から３年を経過した日のいずれか遅い日までの期間■ At least the period of five years from the date of reporting on the termination of the study or the period of three years from the reported date on the final public announcement of the results of the study, whichever is later  □その他（ ）□ Other () | | | |

**個Pieces** **人Man** **情Sentiment** **報A report** **のof** **取Take** **扱Treatment** **い（柏病院）Yes** **(Kashiwa Hospital)**

| **Ⅰ匿名化の状況Ⅰ Status of anonymization** | **匿名化（対応表の作成）の有無Presence or absence of anonymization (creation of correspondence table)** | | | |
| --- | --- | --- | --- | --- |
|  | ■匿名化を行う■ carry out the anonymous  ⇒対応表の作成有無⇒ Create correspondence table  ■ 作成する■ Create  □ 作成しない□ Do not create  □ その他（ ）□ Other ()  □匿名化を行わない（理由： ）□ Do not anonymize (Reason:) | | | |
| **Ⅱ対応表の管理Ⅱ Management of correspondence table**  ㊟対応表の管理・廃棄責任者は個人情報管理 Management and disposal of correspondence table Person in charge  管理者Administrator | **②②**     **対応表の管理Management of correspondence table** | | | |
|  | 個人情報分担Distribution of personal information  管理者Administrator | 所属：救急医学講座Affiliation: Emergency Medical Course  氏名：光永敏哉Name: Toshiya Mitsunaga | 個人情報管理Personal information management  補助者Assistant  （匿名化作業）(Anonymization work) | 所属：救急医学講座Affiliation: Emergency Medical Course  氏名：長谷川意純Name: Tsunemasa Hasegawa |
|  | **④④**    **対応表の保管場所（Storage** **location of** **correspondence table** **(**   救急部スタッフルーム内**）**Emergency department staff room **)** | | | |
|  | 保管方法Storage method  □外部と切り離されたPCを使用して、外部記憶媒体に保存し、□ Save it to an external storage medium using a PC separated from the outside,  鍵の掛かる保管庫に厳重に保管するKeep it strictly in the key storehouse.  ■紙媒体に記録し、鍵の掛かる保管庫に厳重に保管する■ Record it on paper medium and store it strictly in the key storehouse  □その他（ ）□ Other () | | | |
|  | **⑤⑤**           **対応表の廃棄方法Disposal method of correspondence table** | | | |
|  | □外部記憶装置媒体内データを消去した上で、媒体を物理的に破壊□ External storage device Physically destroy the medium after erasing the data in the medium  ■個人情報に注意しシュレッダーで裁断■ Cut by shredder with careful attention to personal information  □その他（ ）□ Other () | | | |
| **Ⅲ情報の管理Ⅲ** **Management of** **information**    ㊟情報の管理責任は研究代表者管理 Management responsibility for information is researcher | **③③**        **保管場所Storage location** **（(** 　 救急医学講座医局内**）**Emergency medical course within the doctor **)** | | | |
|  | 保管方法Storage method  □外部と切り離されたPCを使用して、外部記憶媒体に保存し、鍵の掛かる保管□ Save using a PC detached from the outside to an external storage medium, keep locked  庫に厳重に保管Strictly kept in storage  ■外部と切り離されたPCで、ログイン時及びファイル開封時にﾊﾟｽﾜｰﾄﾞによりｱｸｾｽ制限の掛かったPC内のファイルに記録■ A PC detached from the outside records it in a file in the PC where access is restricted by password when logging in and opening the file  □カルテ内□ In the medical record  □その他（）□ Other ( ) | | | |
|  | **④④**        **廃棄方法Disposal method** | | | |
|  | □外部記憶装置媒体内データを消去した上で、物理的に破壊□ Physically destroy data after deleting data in the external storage device medium  ■PC内のデータ消去ソフトを使用し消去■ Use data erasure software in PC to erase  □その他（ ）□ Other () | | | |
| **Ⅳ安全管理措置IV Safety management measures** | ■ﾊﾟｽﾜｰﾄﾞ設定によりｱｸｾｽ権保有者以外はPCへのｱｸｾｽ不能（ｱｸｾｽ制御）■ other than the access rights holders by the password setting is inaccessible to the PC (access control)  （ｱｸｾｽ権保有者：光永敏哉）( Access right holder: Toshiya Mitsunaga )  ■不正ソフトウェア防止ソフト導入済みのPCを使用■ use unauthorized software prevention software already introduced PC  ■外部との接続の無いPCを使用■ Use a PC without external connection  □入退室管理の実施□ Implement entry control  ■盗難・紛失の防止（□鍵の掛かるキャビネットへの保管 ■持ち歩きの厳禁）■ Prevention of theft and loss (□ storage in a cabinet where keys are hooked ■ prohibition of carrying around)  □その他（）□ Other ( )  ） | | | |
| **ⅤⅤ** **情報（対応表含む）の保管期間Storage period of information (including correspondence table)** | ■少なくとも、当該研究の終了について報告された日から５年を経過した日又は当該研究の結果の最終の公表について報告された日から３年を経過した日のいずれか遅い日までの期間■ At least the period of five years from the date of reporting on the termination of the study or the period of three years from the reported date on the final public announcement of the results of the study, whichever is later  □その他（ ）□ Other () | | | |

**試料・情報の授受に関する記録の作成及び保管についてAbout creation and storage of records concerning sample / information transfer**

本研究において、共同研究機関／試料・情報の提供のみを行う者との間で取り扱う試料・情報の授受の記録作成及び保管については、以下のように定める。In this research, the creation and storage of records and exchanges of samples and information handled with a person who only provides collaborative research institutions / samples / information will be defined as follows. 尚、各共同研究機関及び各機関の研究責任者／試料・情報の提供のみを行う者とその所属機関の一覧については、研究計画書のとおり。As for the list of each collaborative research institution and the research director / instructor only for providing samples / information and the affiliated organizations of each institution, as stated in the research plan.

| **提供元となる機関での取扱いTreatment at the source organization** | | **提供を受ける機関での取扱いTreatment at the receiving organization** | |
| --- | --- | --- | --- |
| **①提供する情報①** **Information to be provided** | ●スコアリングに必要な情報：①意識状態、②脈拍数、③呼吸数、④収縮期血圧or MAP、⑤体温、⑥SpO2、⑦酸素投与、⑧年齢● information necessary for the scoring: ① state of consciousness, ② pulse rate, ③ respiratory rate, ④ systolic blood pressure or MAP, ⑤ body temperature, ⑥ SpO2, ⑦ oxygen administration, ⑧ age  ●その他患者情報：①来院月、②来院時刻、③年齢、④性別、⑤診断カテゴリー（総合内科・呼吸器内科・循環器内科・消化器肝臓内科・神経内科・血液腫瘍内科・糖尿病代謝内分泌内科・腎臓高血圧内科・膠原病内科・外科・整形外科・脳神経外科・形成外科・泌尿器科・耳鼻咽喉科・その他）、⑥転帰**（帰宅・一般床入院・ICU入院・死亡）、⑦採血項目（CRP・アルブミン値・血液ガス）**● Other Patient Information: ① Visit Month, ② Visit Time, ③ Age, ④ Gender, ⑤ Diagnosis Category (Comprehensive Internal Medicine · Internal Medicine of Cardiovascular Medicine · Internal Medicine of Gastroenterology · Internal Medicine of Gastroenterology · Internal Medicine of Neurological Diseases · Internal Medicine of Hematology · Diabetes Metabolism Endocrine Internal Medicine · Kidney Hypertension Internal Medicine · Collagen Disease Internal Medicine · Surgery · Orthopedic Surgery · Neurosurgery · Plastic Surgery · Urology · Otolaryngology · Others), ⑥ Outcome **(Home / General Hospitalization ·** **ICU** **Hospitalization · Death),** **⑦** **Blood Collection Item (CRP · Albumin value · Blood gas)** | **⑫提供を受ける情報（項目）⑫ Information to be provided (item)** | ①①          に同じSame as |
| **②情報の取得経緯②** **Background of information acquisition** | 診療過程で取得されるものである。It is acquired in the medical treatment process. | **⑬提供元機関での情報の取得経緯⑬ History of information acquisition at the providing agency** | ②に同じSame as ② |
| **③提供する情報の由来者③ Origin of information to be provided** | 対応表を作成し、匿名化を施したものを提供する。We prepare a correspondence table and provide what we have anonymized. 但し、対応表の提供は伴わない。However, there is no provision of a correspondence table. | **⑭提供を受ける情報の由来者⑭ The origin of the information to be provided** | 提供を受けた情報そのものを保管。Keep the information itself received . |
| **④提供記録の作成時期④ Time to prepare provision record** | 継続審査または終了時に一括で作成Created in bulk at the ongoing review or at the end | **⑮収受記録の作成時期⑮ When to create receipt records** | 継続審査または終了時に一括で作成Created in bulk at the ongoing review or at the end |
| **⑤提供記録の記録媒体⑤ Recording medium of provision record** | 救急医学講座に設置された管理用PC内の電子ファイルElectronic file in management PC installed in emergency medical course | **⑯収受記録の記録媒体⑯ Recording medium of receipt record** | 千葉大学予防医学センターオミクス応用解析研究室内に設置された管理用PC内の電子ファイルChiba University Preventive Medicine Center Omics Application analysis analysis Electronic file in management PC installed in the laboratory |
| **⑥提供記録の作成者⑥ Creator of provision record** | 東京慈恵会医科大学 救急医学講座 光永敏哉（助教）Tokyo Jikei University School of Medicine Emergency Medicine Course Toshiya Mitsunaga (Assistant Professor) | **⑰収受記録の作成者⑰ Creator of receipt record** | 千葉大学 予防医学センター 関根章博（教授）Chiba University Preventive Medicine Center Sekine Hiroshi (Professor) |
| **⑦提供記録の代用書類⑦ Substitution document of provision record** | 無Nothing | **⑱収受記録の代用書類有無⑱ Substitution document of receipt record** | 無Nothing |
| **⑧提供記録の保管場所⑧** **storage location provides record** | 東京慈恵会医科大学 救急医学講座内の記録管理用キャビネットRecords management cabinet in Tokyo Jikei Medical University emergency medical course | **⑲収受記録の保管場所❺ Storage location of receipt records** | 千葉大学予防医学センターオミクス応用解析研究室内記録管理用キャビネットChiba University Preventive Medicine Center omics applied analysis laboratory records management for cabinet |
| **⑨対応表の管理責任者・管理場所⑨ Person responsible for management of correspondence table · Management place** | 研究個人情報分担管理者の下管理する。Research under the management of individual information sharing administrator .  紙媒体に記録し、鍵のかかる保管庫に厳重に保管する。Record it on a paper medium and store it strictly in a key store. | **⑳提供を受ける機関・責任者機関 Organization / person responsible for receiving offer** | 千葉大学予防医学センター 関根章博（教授）Chiba University Preventive Medicine Center Akihiro Sekine (Professor) |
| **⑩提供先機関・責任者⑩ Destination organization / responsible person** | 東京慈恵会医科大学 救急医学講座 光永敏哉（助教）Tokyo Jikei University School of Medicine Emergency Medicine Course Toshiya Mitsunaga (Assistant Professor) | **㉑提供元機関・責任者㉑ Source organization / responsible person** | 千葉大学 予防医学センター 関根章博（教授）Chiba University Preventive Medicine Center Sekine Hiroshi (Professor) |
| **⑪提供記録の保管期間⑪ Storage period of provided records** | 少なくとも提供予定日（2018年8月）から3年。It is at least three years from the expected offer date (August 2018). | **㉒収受記録の保管期間保 Storage period of receipt records** | 少なくとも本研究の終了報告（２０２０年3月末予定）から3年。At least three years from the end report of this study (scheduled for March 2020 ) . |

1
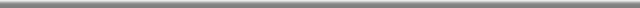

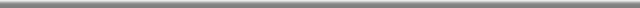

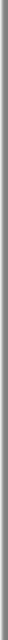

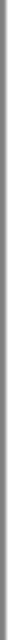

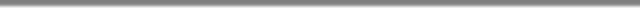

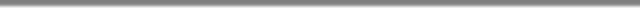

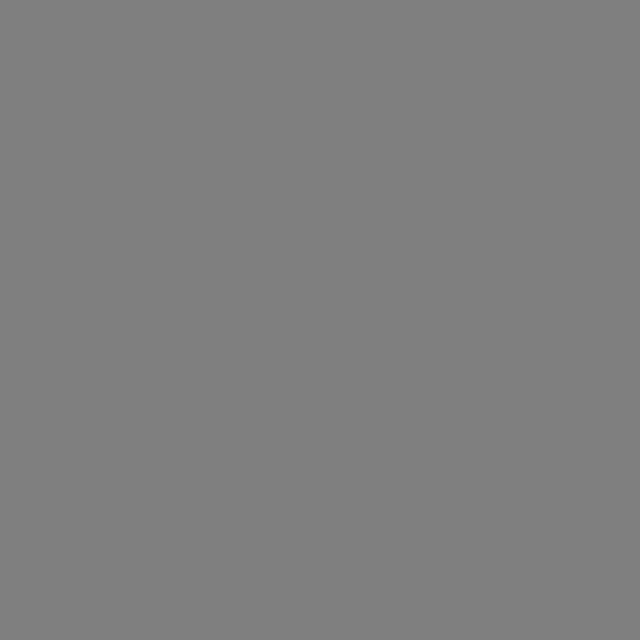

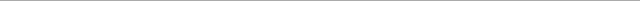

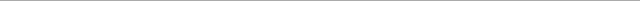

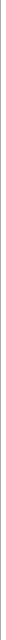

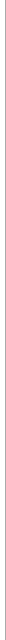

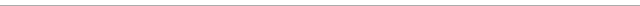

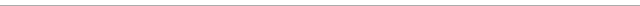

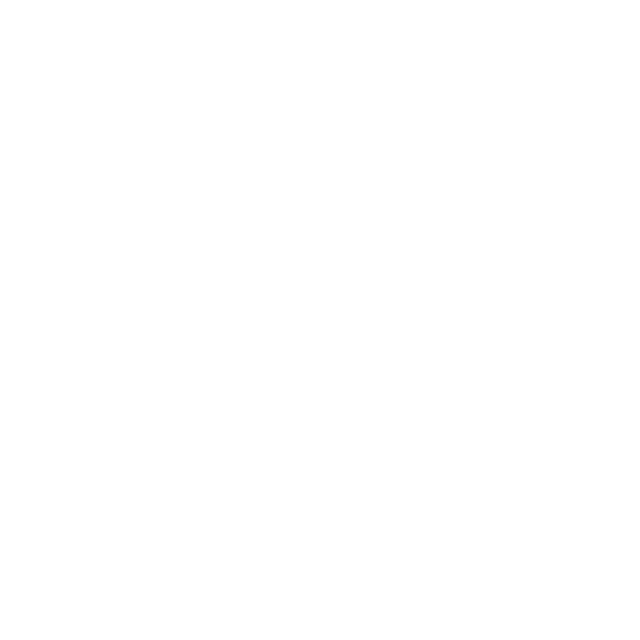


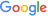


**日本語の原文テキスト:**

長谷川 意純 東京慈恵会医科大学 救急医学講座 助教 データ収集担当者


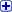
翻訳を改善する
